# BMP9‐induced vascular normalisation improves the efficacy of immunotherapy against hepatitis B virus‐associated hepatocellular carcinoma

**DOI:** 10.1002/ctm2.1247

**Published:** 2023-05-02

**Authors:** Yulong Han, Qiuzhong Pan, Zhixing Guo, Yufei Du, Yaojun Zhang, Yingying Liu, Jingjing Zhao, Jinfeng Xu, Jieying Yang, Dijun Ouyang, Yan Tang, Qijing Wang, Yongqiang Li, Jia He, Mengjuan Yang, Hao Chen, Chaopin Yang, Xinyi Yang, Jinqi You, Yuanyuan Chen, Minghao Ren, Yao Zhu, Jianchuan Xia, Tong Xiang

**Affiliations:** ^1^ Department of Experimental Research State Key Laboratory of Oncology in South China Collaborative Innovation Center for Cancer Medicine Guangdong Key Laboratory of Nasopharyngeal Carcinoma Diagnosis and Therapy Sun Yat‐sen University Cancer Center Guangzhou Guangdong P. R. China; ^2^ Department of Ultrasound Sun Yat‐sen University Cancer Center Guangzhou Guangdong P. R. China; ^3^ Department of Hepatobiliary Surgery Sun Yat‐sen University Cancer Center Guangzhou Guangdong P. R. China; ^4^ Department of Ultrasonography Shenzhen Medical Ultrasound Engineering Center Shenzhen People's Hospital Second Clinical Medical College of Jinan University First Clinical Medical College of Southern University of Science and Technology Shenzhen Guangdong P. R. China; ^5^ Application Business Division Shandong Qilu Stem Cell Engineering Co., Ltd. Shandong Guangdong P. R. China; ^6^ Department of Materials Science Shenzhen MSU‐BIT University Shenzhen Guangdong P. R. China

**Keywords:** BMP9, HBV‐associated HCC, immunotherapy, vascular normalisation

## Abstract

**Background:**

In the past decade, the field of tumour immunotherapy has made a great progress. However, the efficacy of immune checkpoint blocking (ICB) in the treatment of hepatocellular carcinoma (HCC) remains limited. Cytotoxic lymphocyte trafficking into tumours is critical for the success of ICB. Therefore, additional strategies that increase cytotoxic lymphocyte trafficking into tumours are urgently needed to improve patient immune responses.

**Methods:**

Paired adjacent tissue and cancerous lesions with HBV‐associated HCC were subjected to RNA‐seq analysis. Bone morphogenetic protein (BMP9), which reflects vessel normalisation, was identified through Cytoscape software, clinical specimens and Gene Expression Omnibus (GEO) datasets for HCC. The functional effects and mechanism of BMP9 on the tumour vasculature were evaluated in cells and animals. An ultrasound‐targeted microbubble destruction (UTMD)‐mediated BMP9 delivery strategy was used to normalise the vasculature and evaluate therapeutic efficacy mediated by cytotoxic lymphocytes (NK cells) in combination with a PD‐L1 antibody in human cancer xenografts of immune‐deficient mice.

**Results:**

We discovered that hepatitis B virus (HBV) infection‐induced downregulation of BMP9 expression correlated with a poor prognosis and pathological vascular abnormalities in patients with HCC. BMP9 overexpression in HBV‐infected HCC cells promoted intra‐tumoural cytotoxic lymphocyte infiltration via vascular normalisation by inhibiting the Rho‐ROCK‐myosin light chain (MLC) signalling cascade, resulting in enhanced efficacy of immunotherapy. Furthermore, UTMD‐mediated BMP9 delivery restored the anti‐tumour function of cytotoxic lymphocytes (NK cells) and showed therapeutic efficacy in combination with a PD‐L1 antibody in human cancer xenografts of immune‐deficient mice.

**Conclusions:**

HBV‐induced BMP9 downregulation causes vascular abnormalities that inhibit intra‐tumoural cytotoxic lymphocyte infiltration, providing a rationale for developing and combining immunotherapy with BMP9‐based therapy to treat HBV‐associated HCC.

## Graphical abstract

HBV infection was correlated with suppression of BMP9 expression and caused abnormal vasculature in HCC. BMP9 overexpression in HBV‐infected HCC cells could promote intra‐tumoural infiltration of cytotoxic lymphocytes via vascular normalisation by inhibiting the Rho‐ROCK‐MLC pathway, resulting in enhanced anti‐PD‐L1 immunotherapy. UTMD‐mediated BMP9 delivery restored anti‐tumour function and showed therapeutic efficacy of cytotoxic lymphocytes in combination with a PD‐L1 inhibitor.

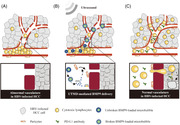

## 1 INTRODUCTION

Hepatocellular carcinoma (HCC) is the most common type of primary liver cancer and is estimated to be the sixth most commonly diagnosed cancer and the third leading cause of cancer‐related death worldwide.^1^ Among the many risk factors for HCC, the most prevalent is viral infection. Approximately 80% of cases worldwide can be attributed to viral infections, with chronic hepatitis B virus (HBV) infection considered to be the dominant type.^2^ Although part of the early‐stage disease may be treated by resection, liver transplantation or ablation, but most patients present have a poor prognosis and unresectable disease.^3^ Given the success of atezolizumab plus bevacizumab therapy, the FDA approved the drug as a first‐line treatment for unresectable liver cancer based on the results of a Phase III clinical trial,^4^ a large number of studies are currently investigating immune checkpoint blockade (ICB) immunotherapy in patients with HCC. However, although ICB has shown benefits in some patients with unresectable HCC who have generally acceptable adverse event profiles, their response rates (approximately 20%) have been unsatisfactory.^5^ Thus, new approaches that potentially improve the clinical benefits achieved with ICB in patients with HCC are urgently needed.

A fundamental prerequisite for the success of ICB is the sufficient trafficking of cytotoxic lymphocytes into tumour tissues.^6^ However, the tumour vasculature, which is one of the initial steps of cytotoxic lymphocyte accumulation^7^ in HCC, is often disordered and tortuous with low perivascular coverage, leading to impaired blood flow and delivery of oxygen, nutrients and therapeutics, including immune cells and antibodies.^8^ Vascular normalisation using rational anti‐angiogenic drugs targeting VEGF to develop a more mature vascular phenotype^9^ is being investigated as a strategy to increase drug and oxygen delivery to cancer cells. In fact, a low dose of bevacizumab (3.6 mg/kg per week) was shown to promote vascular normalisation in tumours, which was not achievable with the higher dose (5 mg/kg per week) typically applied in the clinical setting,^10^ but normalisation using VEGF inhibitors was usually short‐lived (lasting for 7−10 days) and the dosage window to achieve normalisation was relatively narrow.^11^ Therefore, to improve the efficacy of ICBs, alternative strategies that can effectively and long‐term stabilise blood vessels in HCC must be developed. The tumour vasculature frequently lacks adequate coverage by pericytes, which are the components of mature blood vessels.^12^ Increasing pericyte coverage has been proposed as a therapeutic option to achieve vascular normalisation.^13^ Notably, bone morphogenetic protein 9 (BMP9), which is encoded by the growth differentiation factor 2 (*GDF2*) gene, is an important mediator of pericyte recruitment and may also be a promising target for the normalisation of tumour vasculature. BMP9 is a newly discovered member that belongs to the transforming growth factor β (TGF‐β) superfamily.^14^ BMP9 stabilises healthy liver cells and helps maintain the polarisation state and function of cells.^15^ Furthermore, evidence suggests that BMP9 may induce vascular quiescence by regulating vascular remodelling in the maturation phase of angiogenesis.^16^ According to recent studies, BMP9 signalling through the activin receptor‐like kinase 1 (ALK1) receptor in endothelial cells promotes the recruitment of perivascular cells,^17^ suggesting that BMP9 may have applications in normalising the tumour vasculature in HCC. However, achieving the efficient and specific delivery of BMP9 into HCC remains a major obstacle. Therefore, the development of a useful approach for targeted delivery will further improve the efficacy of BMP9‐based therapy. Ultrasound‐targeted microbubble destruction (UTMD) has been recognised as an efficient modality for gene and drug delivery. UTMD is a targeted delivery strategy that compared to conventional systemic delivery of drugs, uses an external ultrasound beam focused within a tumour to achieve the targeted drug release. By doing so, only microbubbles passing through the beam interact with the ultrasound energy.^18^

In this study, we report that HBV promotes abnormal tumour blood vessel formation in HCC through the BMP9/Rho/ROCK/myosin light chain (MLC) axis and describe a potential application of UTMD‐mediated BMP9 delivery as a strategy that might enhance the cytotoxicity of clinical immunotherapy toward HBV‐associated HCC.

## 2 METHODS

### 2.1 Cell lines and cell culture

HepG2.2.15 cells were obtained from Shanghai Zhong Qiao Xin Zhou Biotechnology Co., Ltd. (Shanghai, P. R. China). HB611 and Huh6 cells were obtained from Procell Life Science & Technology Co., Ltd. (Wuhan, P. R. China). C166 cells, mouse brain vascular pericytes (MBVPs) and human brain vascular pericytes (HBVPs) were obtained from Wuhan Fine Biotech Co., Ltd. (Wuhan, China). LO2 cells, HepG2 cells and human umbilical vein endothelial cells (HUVECs) were obtained from Professor Jian‐Chuan Xia (Sun Yat‐sen University Cancer Center). HepG2, HepG2.2.15, Huh6, HB611 and C166 cells were cultured in DMEM‐H supplemented with 10% FBS. RPMI‐1640 supplemented with 10% FBS was added to cultured LO2 cells. HUVECs were cultured in endothelial cell culture medium. MBVPs were cultured in pericyte medium‐mouse. HBVPs were cultured in pericyte medium‐human. Maintained in 95% air and 5% CO_2_ at 37°C, all cells were routinely tested and confirmed mycoplasma‐free by PCR. All cell lines were assayed using a PCR‐based method (16S rDNA‐F: 5′‐ACTCC TACGGGAGGCAGCAGTA‐3′, 16S rDNA‐R: 5′‐TGCACCATCTGTCACTCTGTTAACCTC‐3′) and shown to be free of mycoplasma contamination.

### 2.2 Patients and samples

Immunohistochemical and quantitative tissue samples (10 females and 34 males, all patients had a primary tumour) were obtained from patients who underwent primary HCC resection at the Sun Yat‐sen University Cancer Center between 2005 and 2008, which included adjacent non‐cancerous tissues and HCC tumour tissues. None of these patients had received preoperative chemotherapy or radiotherapy. Tissue samples for RNA‐sequencing (RNA‐seq) were obtained from six patients (two females and four males) between 2019 and 2020 who underwent primary HCC resection at the Cancer Center of Sun Yat‐sen University. These patients also received no preoperative chemotherapy or radiotherapy. The Ethics Committee of Sun Yat‐sen University Cancer Center approved the study protocol and written informed consent from each patient was obtained.

### 2.3 Animal studies

All animal experiments were approved by the institutional Animal Care and Use Committee of Sun Yat‐sen University, and all animals were handled in accordance with institutional guidelines. Four‐week‐old female NOD/ShiLtJGpt‐*Prkdc* ^em26^ *Il2rg* ^em26^/Gpt (NCG) mice were purchased from GuangDong GemPharmatech Co., Ltd. Tumour volume (*V*) was measured using callipers throughout the experiment, and calculated with the formula *V* = (length × width^2^)/2. In vivo experiments used four to five mice per group, unless otherwise noted.

### 2.4 Blood vessel perfusion and leakiness

Female NCG mice were subcutaneously inoculated with 2.5 × 10^6^ HepG2, HepG2.2.15‐HBV or HepG2.2.15‐BMP9 cells in the right flank. At 28 day after subcutaneous inoculation, 0.25 mg of FITC‐dextran 40 kDa (4009, Chondrex Inc.) was intravenously injected into the tumour‐bearing mice, and 10 min after injection, the tumours were harvested. Mouse tissue samples were immediately frozen in OCT compound (for 5‐μm serial sections). The leaky vessel percentage was measured as the percent of dextran that did not co‐localize with CD31‐positive vessels (ImageJ software). In another experiment, 2.5 × 10^6^ HepG2, HepG2.2.15‐HBV or HepG2.2.15‐BMP9 cells were inoculated subcutaneously into the right flanks of female NCG mice. At 28 days after subcutaneous inoculation, the animals were deeply anesthaetised (sodium pentobarbital; 50 mg/kg. injected i.p.) and 0.05 mg of fluorescein‐labelled *Lycopersicon esculentum* (tomato) lectin (FL‐1171‐1, Vector Laboratories) was injected. After injection, the heart continued to beat for around 3 min, and then 10 mL of PBS and 20 mL of 4% paraformaldehyde were perfused through the left ventricle of the animal. After 3 min, the injected tumours were harvested. The perfused vessels percentage was measured as the percent of CD31‐positive vessels that co‐localized with lectin (ImageJ software).

### 2.5 Hypoxia assay

HepG2, HepG2.2.15‐HBV and HepG2.2.15‐BMP9 cells (2.5 × 10^6^) were inoculated subcutaneously into the right flank of female NCG mice after every 7 days to measure tumour size. At 14, 21 and 28 days after subcutaneous inoculation, 60 mg/kg pimonidazole hydrochloride was injected into mice in each tumour‐bearing mouse group (1 h after injected harvested tumours), and paraffin tumour tissue sections were immunostained following the manufacturer's instructions.

### 2.6 Ultrasound imaging of tumour vessel perfusion

Conventional ultrasound (US) and contrast‐enhanced ultrasound (CEUS) images were acquired with an Acuson Sequoia system (Siemens Healthineers, CA) equipped with a linear probe transducer (10L4) to detect perfusion. HepG2, HepG2.2.15‐HBV or HepG2.2.15‐BMP9 cells (2.5 × 10^6^) were inoculated subcutaneously into the right flank. The mice were fed for 30 days and fasted for 12 h before detection. The mouse limbs were pressed and coated with a coupling agent to bring the probe and skin into close contact. The size and echogenicity of each tumour on grayscale US images were recorded. CEUS was performed with a low mechanical index (mainly 0.15−0.19) after the administration of a 0.1‐mL bolus of SonoVue (Bracco Imaging, Milan, Italy) via an intravenous tail vein injection. After contrast agent administration, we continuously recorded images on cine clips for 20 s immediately.

### 2.7 Construction of BMP9‐loaded microbubbles

DSPC, DSPE‐PEG 2000 and stearic‐PEI600 (molar ratio of 82:9:9) were dissolved in a mixture of chloroform and anhydrous methanol to prepare BMP9‐loaded microbubbles. The solution was stirred with a magnetic stirrer for half an hour. Afterward, the organic solvent was removed under vacuum at 60°C using a speed rotary evaporator for 2 h. The remaining organic solvents were further dried under vacuum for 2 h. Then, 5 mL of PBS were added to the formed phospholipid film, which was hydrated in a water bath at 60°C for 15 min. The obtained solution was ultrasounded in a water bath for 2 min and packaged in clean vials to replace the air with perfluoropropane (C3F8). After mechanical oscillation for 30 s and three rounds of centrifugation, cationic microbubbles were obtained. As a method to obtain BMP9 protein‐loaded microbubbles by electrostatic adsorption, de‐ionized water containing 20 μg of BMP9 was incubated with the cationic microbubbles for 15 min and centrifuged three times to remove any unloaded BMP9. For comparison, blank microbubbles were also synthesized.

### 2.8 Statistical analysis

All experiments were performed at least twice. All experiments used Tukey's multiple comparison test and one‐way ANOVA for statistical analyses. Unless specified otherwise, data conforming to a normal distribution from two groups were compared using the Student's *t*‐test. If data from the two groups had a non‐normal distribution or exhibited heterogeneity of variance then the Mann–Whitney U test was used. Tukey's multiple comparison test was suitable for pairwise comparisons between groups. Unless specified otherwise, data conforming to a normal distribution and exhibiting homogeneity of variance from multiple groups were analysed using Dunnett's *t*‐test. If data from multiple groups had a non‐normal distribution or exhibited heterogeneity of variance, the Kruskal–Wallis H test was used. All graphs show the mean value ± SD. Detailed descriptions of methods are available in the Supporting Information.

## 3 RESULTS

### 3.1 Downregulation of BMP9 indicates a poor prognosis for patients with HBV‐associated HCC

**FIGURE 1:**
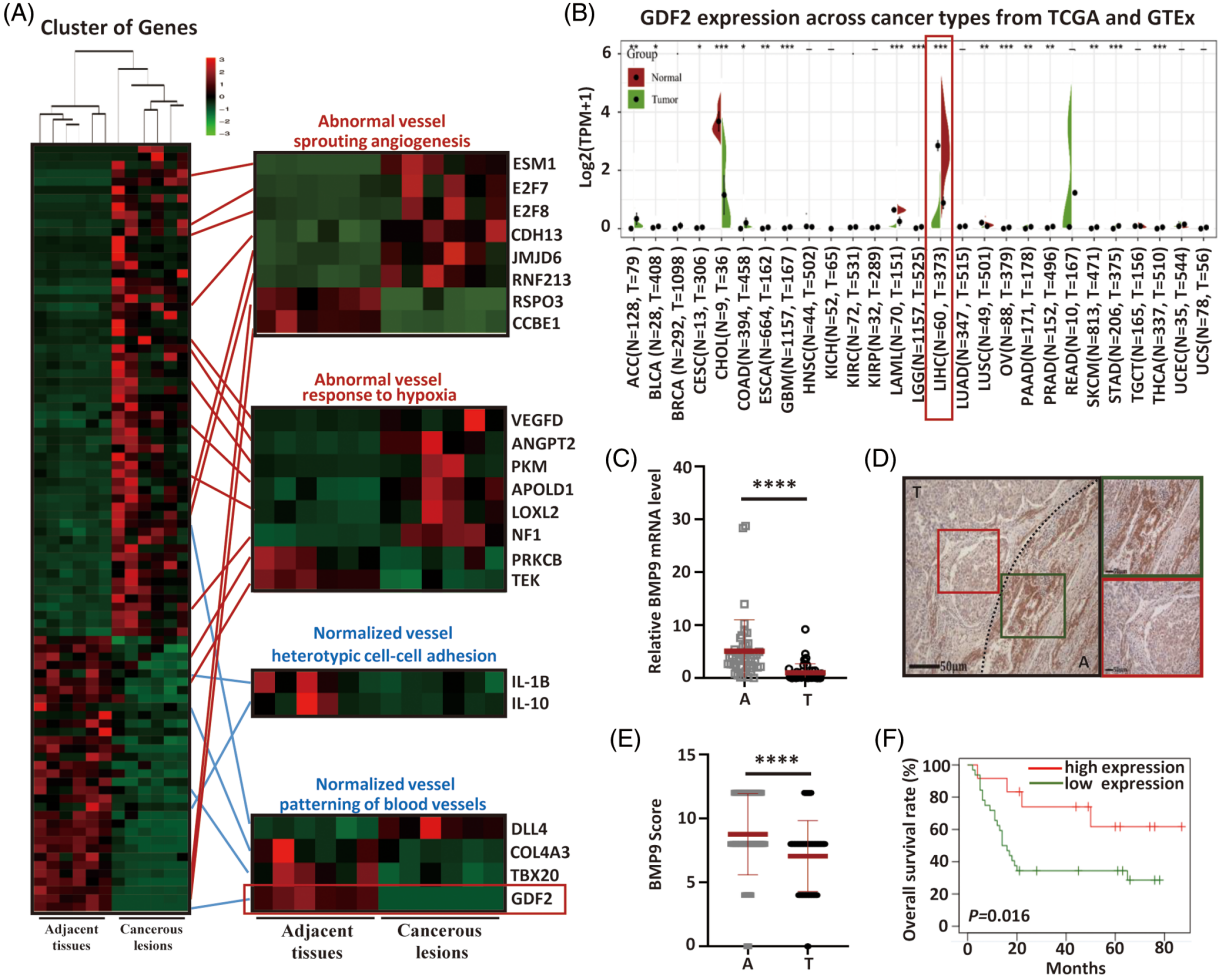
Enrichment of angiogenesis‐related genes and an assessment of overall survival related to BMP9 in patients with HBV‐associated HCC. (A) FPKM values of angiogenesis‐related genes in paired adjacent tissue and cancerous lesions from six patients with HBV‐associated HCC. *p*adj < .05. (B) GDF2 expression across cancer types from TCGA and GTEx (http://sangerbox.com). (C) Relative BMP9 mRNA expression in HCC clinical specimens determined using real‐time PCR. Mean ± SD, *n* = 44, ****p < .0001, Mann–Whitney U test. (D) Representative images of BMP9 expression in HCC clinical specimens visualized using IHC. (E) IHC scores for BMP9 expression in HCC clinical specimens. Mean ± SD, *n* = 23, ****p < .0001, Mann–Whitney U test. (F) The prognostic value of BMP9 in patients with HCC which cut‐off point is the median value (0.02) of the real‐time PCR calculated by SPSS. Mean ± SD, *n* = 44. A, adjacent tissue; T, tumour.

We compared the global gene expression profiles of paired adjacent tissue and cancerous lesions from six patients with HBV‐associated HCC using RNA‐seq analysis to better understand the HCC vasculature. Gene cluster analysis showed that a large number of gene expression levels were dysregulated (Supporting Information Figure S1A). Functional profiling of these dysregulated genes using BiNGO, a Cytoscape plug‐in,^19^ suggested that most of the genes were categorized into nine functional groups: location, immune, stimulus, cellular process, metabolism, regulation, development and reproduction (Supporting Information Figure S1B). In the development group, 92 differentially expressed genes participated in angiogenesis. Among these angiogenesis‐related genes, the upregulated genes were mostly related to sprouting angiogenesis^20^ and hypoxia,^21^ which reflect abnormal vessels. In contrast, the downregulated genes were mostly related to heterotypic cell–cell adhesion^22^ and patterning of blood vessels,^23^ which reflect vessel normalisation (Figure 1A), suggesting that the vasculature in patients with HBV‐associated HCC is abnormal and dysfunctional. Among the vessel normalisation‐related genes, the transcript levels of the most significantly downregulated gene, *GDF2*, were further verified using SangerBox (http://sangerbox.com/). Compared to the level detected in normal liver cells, the *GDF2* expression level was reduced in two common liver cancers, liver hepatocellular carcinoma and cholangiocarcinoma, but not in other cancer types (Figure 1B). Because *GDF2* has been reported to stabilise healthy liver cells and help maintain the polarisation state and function of cells,^15^ our data suggest that *GDF2* exerts anti‐oncogenic activities in HCC. *GDF2* encodes the protein BMP9. Decreased expression of BMP9 in patients with HBV‐associated HCC was validated in clinical specimens from Sun Yat‐sen University Cancer Center (Figure 1C,E). Then, we tried to determine the prognostic value of BMP9 in patients with HBV‐associated HCC. We analysed BMP9 levels using the real‐time PCR in 44 patients with HCC (Supporting Information Table S1), and shorter overall survival was significantly associated with low BMP9 expression (Figure 1F). By analysing two different Gene Expression Omnibus (GEO) datasets for HCC, we also found that lower BMP9 mRNA levels were associated with poorer overall survival outcomes in patients with HCC (Supporting Information Figure S1C,D) and that BMP9 expression was consistently downregulated across all grades of HCC (Supporting Information Figure S1E). These data indicate that the downregulation of BMP9 is associated with a poor prognosis in HBV‐associated HCC patients.

### 3.2 Suppression of BMP9 expression correlates with HBV infection

**FIGURE 2:**
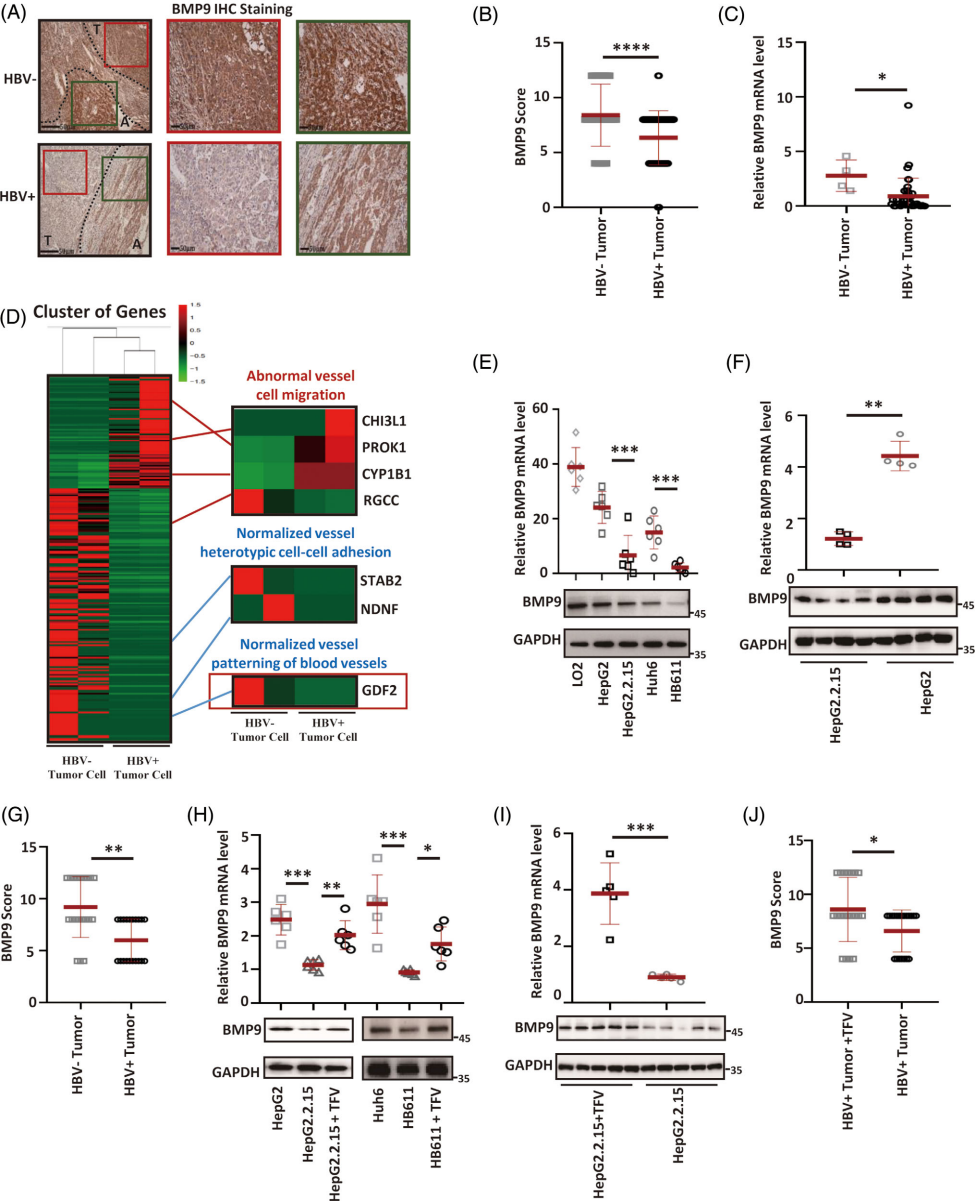
HBV regulates BMP9 expression. (A) Representative images of BMP9 expression in HBV‐uninfected and HBV‐infected HCC samples visualized using IHC. A, adjacent tissue; T, tumour. (B) IHC scores for BMP9 in HBV‐uninfected and HBV‐infected HCC samples (eight HBV‐uninfected patients with HCC, *n* = 40; 15 HBV‐infected patients with HCC, n = 75). The BMP9 score was determined by performing a microscopic analysis of randomly chosen fields at 200× magnification. Mean ± SD, ****p < .0001, Mann–Whitney U test. (C) Relative BMP9 mRNA levels in HBV‐uninfected and HBV‐infected HCC samples (40 HBV‐infected patients with HCC and four HBV‐uninfected patients with HCC). Mean ± SD, *n* = 44, *p < .05, Student's *t*‐test. (D) FPKM values for all differentially expressed genes (DEGs) in two paired HBV‐uninfected and two HBV‐infected HCC tumour cells. *p*adj < .05. (E) Relative BMP9 mRNA (upper panel) and protein (lower panel) levels in HBV‐infected and HBV‐uninfected hepatoma cells. Mean ± SD, *n* = 6, ***p < .001, Student's *t*‐test. (F) Relative BMP9 mRNA and protein levels in xenografts formed by the HepG2.2.15 and HepG2 cell lines. Mean ± SD, *n* = 4, **p < .01, Mann–Whitney U test. (G) IHC scores for BMP9 in xenografts formed by the HepG2.2.15 and HepG2 cell lines. The BMP9 score was determined by performing a microscopic analysis of randomly chosen fields at 200× magnification. Mean ± SD, *n* = 20, **p < .01, Mann–Whitney U test. (H) Relative BMP9 mRNA (upper panel) and protein (lower panel) levels in HBV‐uninfected cells, HBV‐infected cells and HBV‐infected hepatoma cells treated with TFV. Mean ± SD, *n* = 6, ***p < .001, **p < .01 and *p < .05, Dunnett's *t*‐test. (I) Relative BMP9 mRNA and protein levels in xenografts formed by the HepG2.2.15 and HepG2.2.15 cell lines following TFV treatment. Mean ± SD, *n* = 5, ***p < .001, Student's *t*‐test. ( J) IHC scores for BMP9 in xenografts formed by the HepG2.2.15 and HepG2.2.15 cell lines following tenofovir (TFV) treatment. Mean ± SD, *n* = 20, *p < .05, Mann–Whitney U test.

Chronic infection with HBV has been implicated in the occurrence and development of HCC,^24^ and we wondered whether the downregulation of BMP9 in HCC correlates with HBV infection. BMP9 expression was examined in HBV‐uninfected and ‐infected HCC tissues. Interestingly, HBV‐infected HCC tissues expressed lower levels of BMP9 than HBV‐uninfected HCC tissues (Figure 2A‐C). Vessel normalisation‐related genes, including *GDF2*, were also significantly downregulated in tumour cells isolated from HBV‐infected HCC tissues compared with HBV‐uninfected HCC tissues (Figure 2D). We also evaluated the correlation between HBV antigen (HBsAg or HBcAg) and BMP9 expression, a negative correlation pattern was observed between HBV antigen and BMP9 (Supporting Information Figure S2A,B). BMP9 expression was analysed in paired HBV‐infected and ‐uninfected hepatoma cells (HepG2.2.15 vs. HepG2 cells and HB611 vs. Huh6 cells) to further determine whether the downregulation of BMP9 correlated with HBV infection (Supporting Information Figure S2C,D). Real‐time PCR and WB showed that the levels of BMP9 mRNA and protein in HBV‐infected HCC cells were downregulated compared with those in non‐HBV‐infected cells (Figure 2E). HBV‐infected and ‐uninfected hepatoma cells (HepG2.2.15 vs. HepG2 cells) were injected subcutaneously into mice to evaluate BMP9 expression in vivo. At 30 days post‐implantation, the xenografts were dissected and examined for BMP9 expression. Compared with HepG2 cells, HepG2.2.15 cells infected with HBV (Supporting Information Figure S2E) exhibited lower levels of BMP9 expression (Figure 2F,G, Supporting Information Figure S2F,G). In addition, tenofovir, an HBV nucleotide reverse transcriptase inhibitor^25^ (Supporting Information Figure S2H), was used to evaluate the effects of HBV infection on BMP9 expression. Inhibition of HBV in HBV‐infected hepatoma cells (HepG2.2.15 and HB611 cells) and their corresponding xenograft models with tenofovir (TFV) upregulated BMP9 expression (Figure 2H‐J, Supporting Information Figure S2I). However, TFV did not affect BMP9 expression in HBV‐uninfected hepatoma cells (HepG2 and Huh6 cells) (Supporting Information Figure S2J). These data indicate that the downregulation of BMP9 expression in HCC correlates with HBV infection.

### 3.3 Suppression of BMP9 expression by HBV infection is linked to pathological vascular abnormalities

**FIGURE 3:**
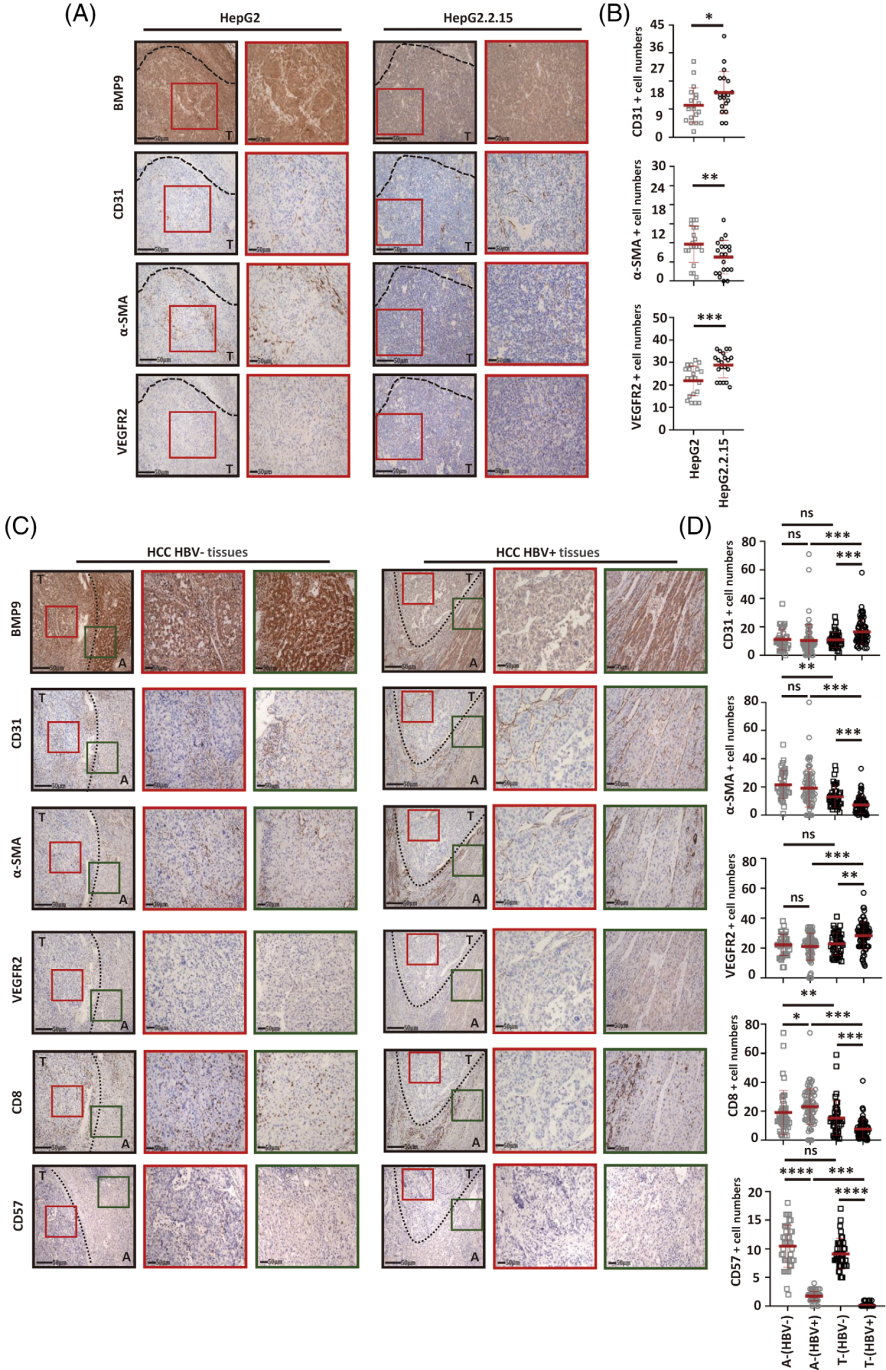
Suppression of BMP9 expression by HBV infection is linked to pathological vascular abnormalities. (A) Serial sections of HepG2 and HepG2.2.15 xenografts were stained with antibodies against human BMP9, mouse CD31 (endothelial cell marker), α‐SMA (mature vessel marker) or VEGFR2 (immature vessel marker). (B) Number of mice CD31^+^, α‐SMA^+^ and VEGFR2^+^ cells based on image J‐Angiogenesis analyser analysis of randomly chosen fields at 200× magnification. Mean ± SD, *n* = 20, *p < .05, **p < .01 and ***p < .001, Student's *t*‐test. (C) Serial sections of HBV‐uninfected and HBV‐positive human HCC tissues were stained with antibodies specific for human BMP9, CD31 (endothelial cell marker), α‐SMA (mature vessel marker), VEGFR2 (immature vessel marker), CD8 (T‐cell marker) or CD57 (natural killer cell marker). (D) Number of human CD31^+^, α‐SMA^+^, VEGFR2^+^, CD8^+^ and CD57^+^ cells determined based on image J‐Angiogenesis analyser analysis of randomly chosen fields at 200× magnification. Mean ± SD, HBV‐ tissues *n* = 40, HBV+ tissues *n* = 75, ns: not significant, *p<.05, **p<.01 and ***p<.001, Kruskal–Wallis H test and Dunnett's *t*‐test. A, adjacent tissue; T, tumour.

BMP9 is an important mediator of pericyte recruitment and potentially represents a promising target for achieving tumour vasculature normalisation. Next, we investigated the correlation between the suppression of BMP9 expression by HBV infection and the formation of the vasculature in xenograft and human HCC tissues. Xenografts derived from HBV‐infected HepG2.2.15 cells showed a reduction in BMP9 expression accompanied by an increase in angiogenesis, which was defined based on CD31 staining, and a decrease in pathological vessel normalisation, which was defined based on the expression of a mature vessel marker (α‐SMA) and an immature vessel marker (VEGFR2), compared with HBV‐uninfected HepG2‐derived tumours (Figure 3A,B). We further investigated the formation of the vasculature in human HCC tissues. As expected, HBV‐infected HCC expressed lower levels of BMP9, accompanied by an increase in angiogenesis and a decrease in pathological vessel normalisation compared with HBV‐uninfected HCC. The development of abnormal vessels leads to impaired delivery of immune cells into tumours. Therefore, we further investigated cytotoxic lymphocytes (CD8^+^ T cells and natural killer [NK] cells) in human HCC tissues, and the number of cytotoxic lymphocytes significantly reduced in HBV‐infected HCC tissues, which expressed BMP9 at lower levels, compared with HBV‐uninfected HCC tissues (Figure 3C,D). These data indicated that BMP9 expression was suppressed by HBV infection in HCC, which might play an important role in the development of the pathologically abnormal vasculature.

### 3.4 BMP9 overexpression inhibits the abnormal vasculature in HBV‐infected HCC

**FIGURE 4:**
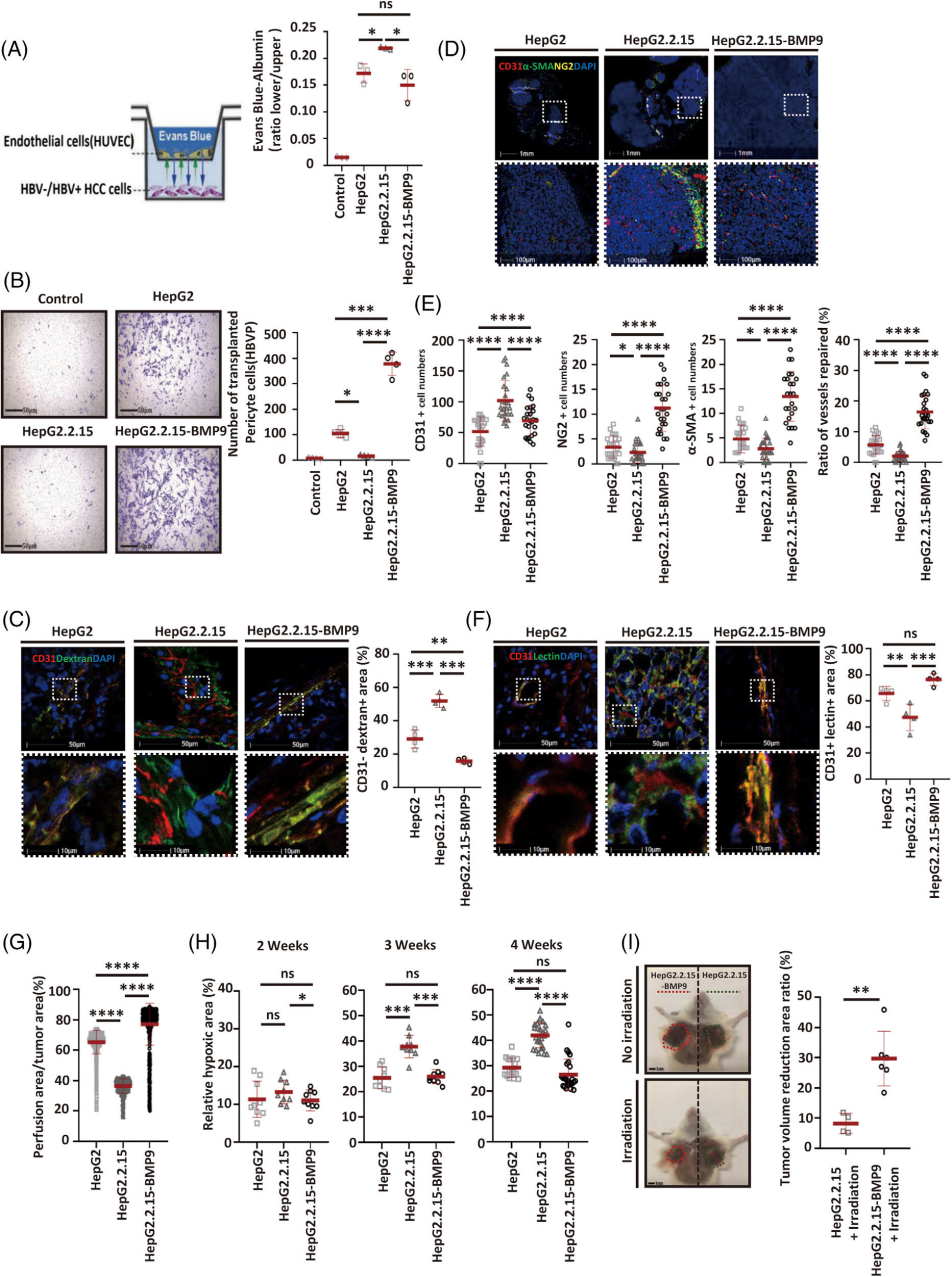
BMP9 overexpression inhibits the abnormal vasculature in HBV‐infected HCC. (A) Working model illustrating the Evans Blue‐albumin permeation assay. Evans Blue‐albumin permeation assay performed with different HCC cell lines. Mean ± SD, *n* = 3, *p < .05, ns: not significant, Kruskal–Wallis H test. (B) Representative images of human brain vascular pericyte (HBVP) migration toward HBV‐infected hepatoma cell lines compared with that toward HBV‐uninfected hepatoma cell lines. Quantification of human brain vascular pericyte (HBVP) migration toward HBV‐infected hepatoma cell lines compared with that toward HBV‐uninfected hepatoma cell lines. Mean ± SD, *n* = 4 *p < .05, ***p < .001, ****p < .0001 and Kruskal–Wallis H test. (C) Representative images (left panel) and quantification (right panel) of dextran and CD31 staining to assess vessel permeability in xenografts formed by different HCC cell lines. The CD31‐dextran+ area/percentage of total dextran+ area were analysed to determine vessel permeability by performing a microscopic analysis of randomly chosen fields at 600× magnification. Mean ± SD, *n* = 4, **p < .01 and ***p < .001, Dunnett's *t*‐test. (D) Representative images of immunofluorescence staining for mouse CD31, α‐SMA and NG2 and DAPI in xenografts formed by different HCC cell lines. (E) Number of immunofluorescence staining for mouse CD31, NG2 (pericyte cell marker), α‐SMA and repaired vessels (CD31 encapsulated by NG2 or α‐SMA indicates a normalised blood vessel) in xenografts formed by different HCC cell lines based on a microscopic analysis of randomly chosen fields at 200× magnification. Mean ± SD, *n* = 25, ns: not significant, *p < .05, ***p < .001 and ****p < .0001, Kruskal–Wallis H test. (F) Representative images (left panel) and quantification (right panel) of lectin and CD31 staining to assess vessel perfusion efficiency in xenografts formed by different HCC cell lines. The lectin+CD31^+^ area/percentage of the total CD31^+^ area was analysed to assess perfusion efficiency by performing a microscopic analysis of randomly chosen fields at 600× magnification. Mean ± SD, *n* = 4, ns: not significant, **p < .01 and ***p < .001, Dunnett's *t*‐test. (G) CEUS detection of the perfusion area of xenografts formed by different HCC cell lines. Mean ± SD, *n* = 486, ****p < .0001, Kruskal–Wallis H test. (H) The percentage of the hypoxic area in tumour sections from xenografts formed by different HCC cell lines was determined by performing a microscopic analysis of randomly chosen fields at 200× magnification. Mean ± SD, *n* = 9 (2 and 3 weeks), *n* = 25 (4 weeks), ns: not significant, *p < .05, ***p < .001 and ****p < .0001, Kruskal–Wallis H test. (I) Representative images (left panel) and quantification (right panel) of the effect of radiotherapy (2 Gy) on xenografts formed by HepG2.2.15 or HepG2.2.15‐BMP9 cells. Mean ± SD, *n* = 6, **p < .01, Student's *t*‐test.

One of the hallmarks of tumour vascular abnormalities is their increased permeability, which is due to the instability of endothelial connections and contributes to the formation of high interstitial pressure and poor blood perfusion of oxygen and nutrients within the tumour.^26^ We assessed whether BMP9 affected the integrity of the tumour vasculature in HBV‐infected HCC by first evaluating the effects of BMP9 on the endothelial junctions of HBV‐infected HCC cells. HUVECs were co‐cultured in transwell inserts in the presence of tumour cells for 96 h, and then Evans blue‐albumin was measured through the endothelial monolayer for 90 min (Figure 4A). In addition, HUVECs were co‐cultured in the presence of tumour cell supernatant for 96 h, and endothelial junctions were demonstrated by VE‐cadherin fluorescent staining. In the lower chamber, when HUVECs were co‐cultured with HBV‐infected HCC cells compared with those co‐cultured with HBV‐uninfected HCC cells, a significant increase in the level of Evans Blue‐albumin was detected. In contrast, when HUVECs were co‐cultured in the presence of BMP9‐overexpressing HBV‐infected HCC cells, compared to HUVECs co‐cultured with HBV‐uninfected HCC cells, there was no increase in Evans Blue‐albumin permeability. Besides, a significant decrease in the level of VE‐cadherin expression was detected when HUVECs were co‐cultured with HBV‐infected HCC cell supernatant compared with those co‐cultured with HBV‐uninfected HCC cell supernatant. In contrast, when HUVECs were co‐cultured in the presence of BMP9‐overexpressing HBV‐infected HCC cell supernatant, a significant increase in the level of VE‐cadherin expression was observed compared to HUVECs co‐cultured with HBV‐uninfected HCC cell supernatant. (Figure 4A, Supporting Information Figure S3A), suggesting that BMP9 prevents the destabilisation of endothelial junctions triggered by HBV. Pericytes are crucial for vessel maturation, and a tumour vasculature with high permeability is characterised by pericyte insufficiency.^27^ We also observed a decrease in pericyte migration toward HBV‐infected HCC cells compared with that toward HBV‐uninfected HCC cells. In contrast, in the presence of BMP9‐overexpressing HBV‐infected HCC cells, no decrease was observed compared with HBV‐uninfected HCC cells (Figure 4B). Consistent with this hypothesis, xenografts derived from HBV‐infected HCC cells showed extensive leakage of intravenously administered FITC‐dextran compared with those derived from HBV‐uninfected HCC cells; conversely, xenografts derived from BMP9‐overexpressing HBV‐infected HCC cells exhibited an absence of leakage (Figure 4C). The decreased permeability of the tumour vessels in xenografts derived from BMP9‐overexpressing HBV‐infected HCC cells was consistent with the increased levels of mature vessel markers (α‐SMA and NG2) detected in these vessels (Figure 4D,E). Next, we evaluated the blood perfusion of tumour vasculature by intravenous administration of biotinylated tomato lectin, which highlights the vascular lumen. Blood perfusion was markedly reduced in HBV‐infected HCC cell xenografts compared to HBV‐uninfected HCC cell xenografts, while BMP9 overexpression negated the decrease in vessel perfusion (Figure 4F). Furthermore, CEUS showed that the perfusion images and intra‐tumoural signal intensity of the maximum section of tumours were higher in xenografts derived from BMP9‐overexpressing HBV‐infected HCC cells than in those derived from HBV‐infected HCC cells or HBV‐uninfected HCC cells (Figure 4G, Supporting Information Figure S3B,C). This increased tumour vessel perfusion coincided with decreased levels of tumour hypoxia in BMP9‐overexpressing HBV‐infected HCC cell xenografts (Figure 4H, Supporting Information Figure S3D). Since abnormal vascular function in tumours may reduce the effectiveness of radiotherapy, we tested how BMP9 affected the efficacy of local irradiation in HBV‐infected HCC cell xenografts. In the BMP9 overexpression group, a single dose of radiation (2 Gy) resulted in a 30% decrease in tumour volume compared with the volume in the untreated group at 7 days post‐irradiation. In comparison, only a 10% reduction was observed in control mice (Figure 4I, Supporting Information Figure S3E). Altogether, these data suggest that BMP9 prevents the tumour endothelial destability in HBV‐infected HCC.

### 3.5 BMP9 inhibits the abnormal vasculature by targeting the Rho‐ROCK‐MLC pathway

**FIGURE 5:**
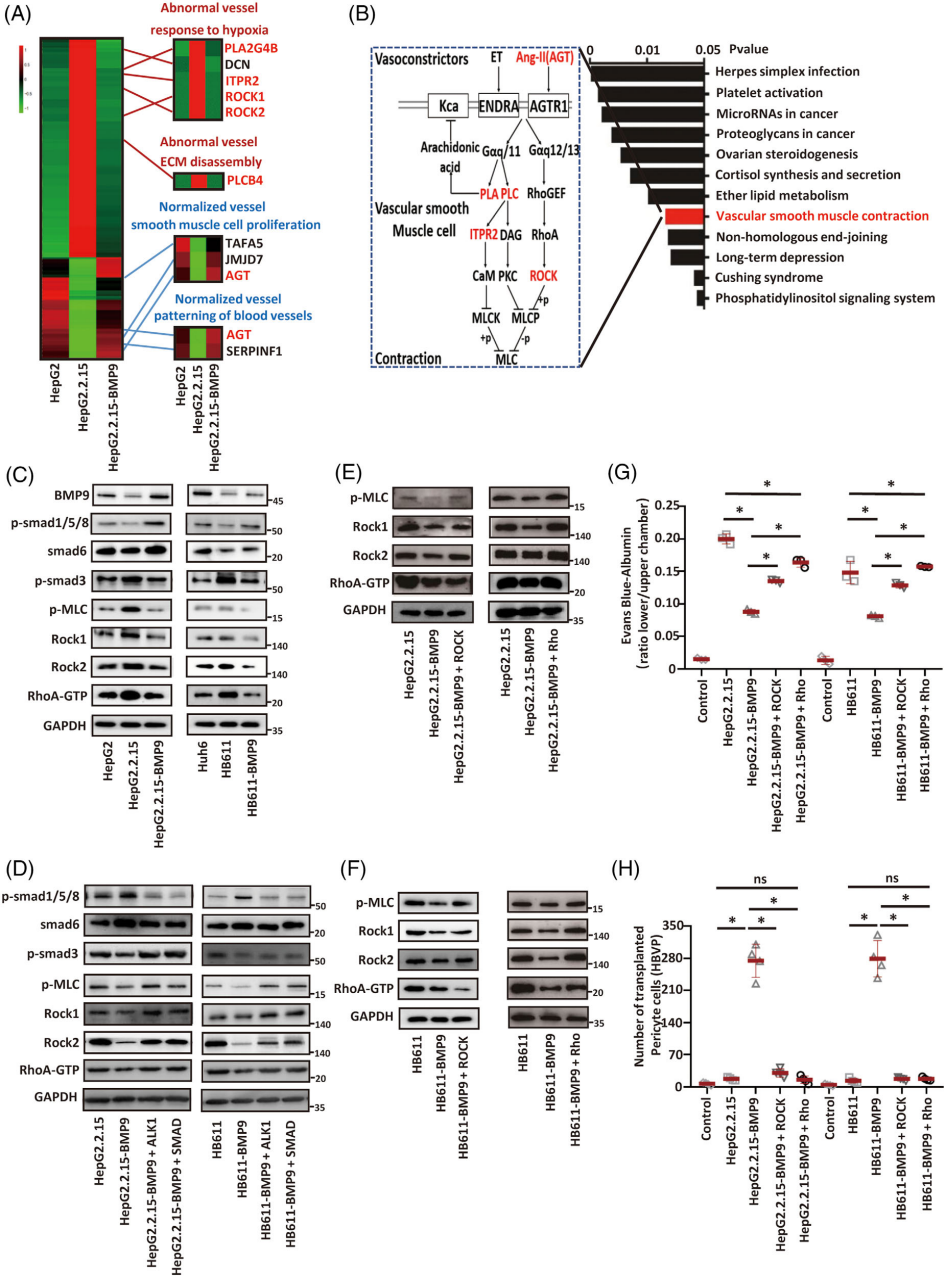
BMP9 promotes the normalisation of the tumour vasculature via the Rho/ROCK/MLC axis. (A) FPKM values of all DEGs in HUVECs pre‐incubated with different HCC cell lines. *p*adj < .05. (B) Enrichment scores for the significant biological processes and image of vascular smooth muscle contraction signalling. (C) Immunoblots of different hepatoma cell lines. The RhoA‐GTP protein was collected using a RhoA activation assay kit (80601) according to the manufacturer's instructions. (D) HBV‐infected hepatoma cell lines overexpressing BMP9 were treated with 25 μM ALK1 inhibitor (HY‐12274) for 2 h or 5 μM smad1/5/8 inhibitor (HY‐12071) for 1 h, followed by western blot analyses. (E, F) HBV‐infected hepatoma cell lines overexpressing BMP9 were treated with 1 unit/mL Rho activator I (CN01) or 50 μM ROCK activator (HY‐W012722) for 30 min, followed by western blot analyses of p‐MLC, Rock1, Rock2, GAPDH and RhoA‐GTP levels. The RhoA‐GTP protein was collected using the RhoA activation assay kit (80601) according to the manufacturer's instructions. (G) Quantification of Evans Blue‐albumin permeation through the upper endothelial monolayer in the lower chamber containing different hepatoma cell lines with or without Rho or ROCK activator stimulation for 24 h. Mean ± SD, *n* = 3, *p < .05, Kruskal–Wallis H test. (H) Quantification of pericyte migration toward different hepatoma cell lines with or without Rho or ROCK activator stimulation for 24 h. Mean ± SD, *n* = 4, ns: not significant, *p < .05, Kruskal–Wallis H test.

We compared the gene expression profiles of HUVECs pre‐incubated with HBV‐uninfected, HBV‐infected or BMP9‐overexpressing HBV‐infected HCC cells (HepG2, HepG2.2.15 and HepG2.2.15‐BMP9 cells, respectively) for 24 h to understand the molecular mechanisms of BMP9‐induced vascular normalisation in HBV‐infected HCC. Six differentially expressed genes reflecting abnormal vessels were consistently upregulated in the HUVECs pre‐incubated with HBV‐infected cells compared with the HUVECs pre‐incubated with HBV‐uninfected or BMP9‐overexpressing cells. In contrast, four differentially expressed genes reflecting vessel normalisation were consistently downregulated in the HUVECs pre‐incubated with HBV‐infected cells compared with the HUVECs pre‐incubated with HBV‐uninfected or BMP9‐overexpressing cells (Figure 5A). Thus, the total genes (4 + 6) may account for the vascular normalisation induced by BMP9. Kyoto Encyclopaedia of Genes and Genomes pathway analysis subsequently revealed that six genes were significantly enriched in the Rho‐ROCK‐MLC signalling pathway in vascular smooth muscle contraction, contributing to the physiological functions of vascular permeability (Figure 5B). Rho/ROCK activation leads to actomyosin‐based contraction and actin cytoskeletal recombination through indirect phosphorylation of MLC (p‐MLC), thereby altering the integrity of endothelial cell tight junctions to affect vascular permeability.^28^ We first investigated the mechanism by which BMP9 inhibits Rho‐Rock‐myosin. On the one hand, BMP9 can bind to ALK‐1 to influence p‐Smad1/5/8 signalling,^29^ which leads to the maturation and stabilisation of newly formed blood vessels.^30^ On the other hand, BMP can effectively attenuate the Rho‐ROCK signalling pathway by inhibiting Smad3 activation^31^ through increasing Smad6^32^ expression, which is a p‐Smad1/5/8‐induced Smad inhibitor.^33^ We hypothesised that BMP9 can regulate the Rho‐ROCK‐MLC through p‐Smad1/5/8‐Smad6‐p‐Smad3 signalling. Western blot analyses of cell lysates harvested from HUVECs pre‐incubated with HBV‐uninfected, HBV‐infected or BMP9‐overexpressing HBV‐infected hepatoma cells were performed. A significant decrease in the p‐Smad1/5/8 and Smad6 levels following the increase in the p‐Smad3 level was detected in the HUVECs pre‐incubated with HBV‐infected hepatoma cells compared with those pre‐incubated with HBV‐uninfected hepatoma cells. Conversely, the decreased levels of p‐Smad1/5/8 and Smad6 following the increased level of p‐Smad3 were abolished in the HUVECs pre‐incubated with BMP9‐overexpressing HBV‐infected hepatoma cells (Figure 5C, Supporting Information Figure S4A). Meanwhile, using a specific antibody against p‐MLC (Ser19) to determine whether BMP9 reduced vascular permeability by decreasing p‐MLC levels. A significant increase in the p‐MLC level was detected in the HUVECs pre‐incubated with HBV‐infected hepatoma cells compared with those pre‐incubated with HBV‐uninfected hepatoma cells. Conversely, the increased levels of p‐MLC were abolished in the HUVECs pre‐incubated with BMP9‐overexpressing HBV‐infected hepatoma cells (Figure 5C, Supporting Information Figure S4A). Alternatively, cell lysates can be directly subjected to western blot analysis to investigate changes in ROCK expression using a specific antibody. As expected, HBV‐infected cells exhibited lower levels of BMP9 expression, leading to increased levels of the RhoA‐GTP and ROCK proteins in HUVECs. The increased levels of RhoA‐GTP and ROCK in HUVECs were abolished by BMP9 overexpression in HBV‐infected hepatoma cells (Figure 5C, Supporting Information Figure S4A). We then treated HUVECs with ALK1 or p‐Smad1/5/8 inhibitor. Both drugs effectively decreased Smad6 expression and increased p‐Smad3‐Rho‐ROCK‐MLC signalling levels in HUVECs pre‐incubated with BMP9‐overexpressing HBV‐infected cells to a level similar to that in HUVECs pre‐incubated with HBV‐infected hepatoma cells (Figure 5D, Supporting Information Figure S4B). Similarly, we treated HUVECs with Rho and ROCK activators.^34^, ^35^ Both drugs effectively increased the p‐MLC level in HUVECs pre‐incubated with BMP9‐overexpressing HBV‐infected cells to a level similar to that pre‐incubated with HBV‐infected hepatoma cells (Figure 5E,F, Supporting Information Figure S4C,D). Furthermore, Rho and ROCK activators suppressed the endothelial junctions and pericyte recruitment activity of BMP9‐overexpressing HBV‐infected hepatoma cells in vitro (Figure 5G,H, Supporting Information Figure S5A‐F). Together, these results indicate that BMP9 can inhibit the Rho‐ROCK‐MLC pathway by p‐Smad1/5/8‐Smad6‐p‐Smad3 signalling in HBV‐infected HCC. Thus, the abnormal blood vessels of the tumour can be inhibited eventually.

### 3.6 Anti‐tumour activity is increased by combination therapy with NK cells and PD‐L1 ICB targeting in BMP9‐overexpressing HBV‐infected HCC

**FIGURE 6:**
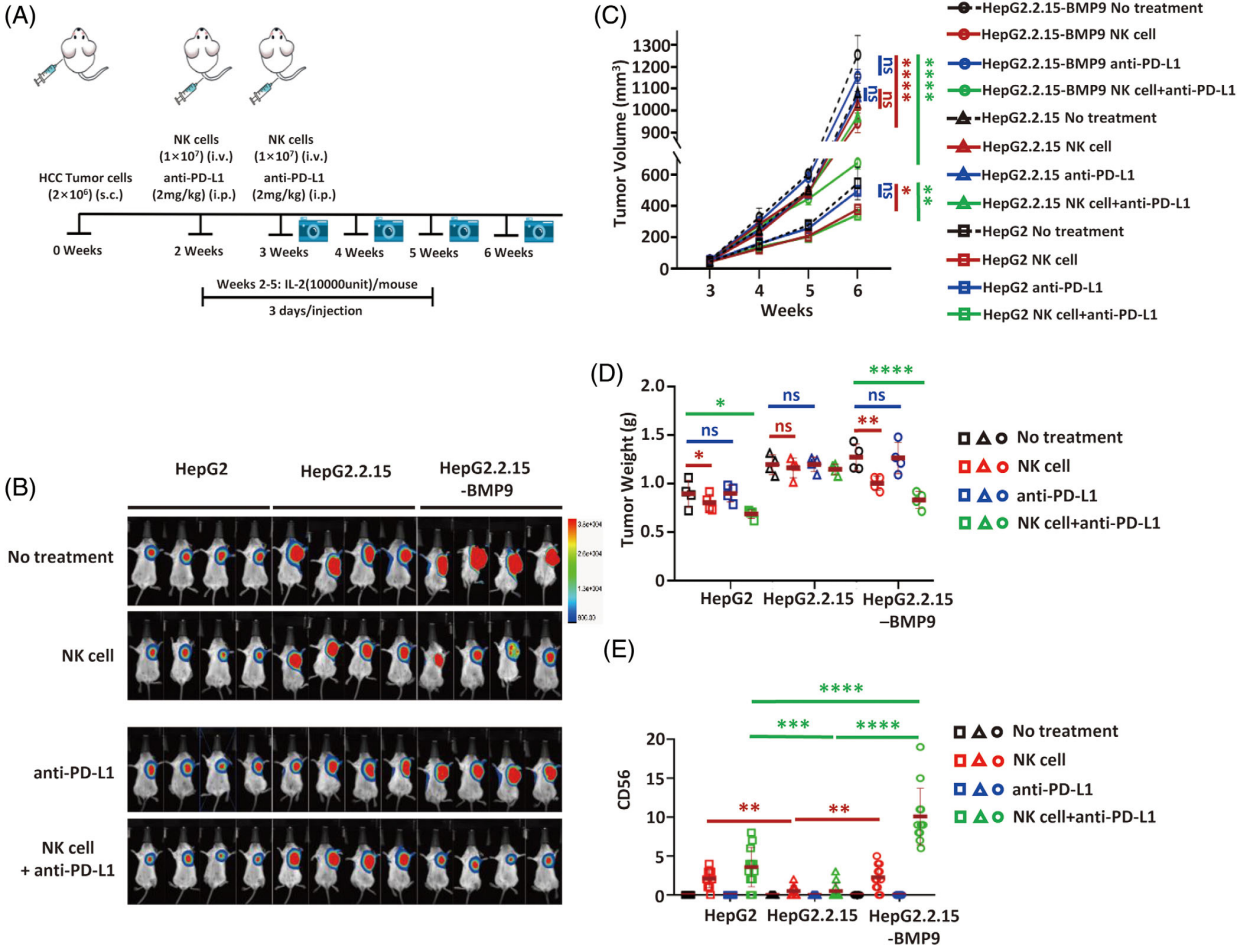
Synergistic anti‐tumour activities of combination therapy with NK cells and PD‐L1 ICB targeting in BMP9‐overexpressing HBV‐infected HCC. (A) Schematic of in vivo studies using NK cells and/or an anti‐PD‐L1 immune checkpoint antibody in mouse xenograft models established with HepG2, HepG2.2.15 or HepG2.2.15 cells overexpressing BMP9. (B) Whole‐body luminescence imaging was used to evaluate xenografts derived from HepG2, HepG2.2.15 or HepG2.2.15 cells overexpressing BMP9 that were infused with or without NK cells and/or the anti‐PD‐L1 immune checkpoint antibody. (C,D) Growth curves (C) and tumour weights (D) of xenografts derived from HepG2, HepG2.2.15 or HepG2.2.15 cells overexpressing BMP9 in mice that were infused with or without NK cells and/or the anti‐PD‐L1 immune checkpoint antibody. Mean ± SD. *n* = 4, ns: not significant, *p < .05, **p < .01 and ****p < .0001, Kruskal–Wallis H and Dunnett's *t*‐test. € Quantification of CD56 staining in xenografts derived from HepG2, HepG2.2.15 or HepG2.2.15 cells overexpressing BMP9 in mice that were infused with or without NK cells and/or the anti‐PD‐L1 immune checkpoint antibody. Mean ± SD, *n* = 4, **p < .01, ***p < .001 and ****p < .0001, Kruskal–Wallis H test.

The human BMP9 protein is highly homologous (approximately 96% amino acid identity) with the mouse BMP9 protein (Supporting Information Figure S6A), and these proteins have highly similar functions. Therefore, we determined whether the effect of BMP9 on vascular normalisation was mediated by promoting the infiltration of immune cells into tumour tissues. Because, on the one hand, NK cells can directly target HBV‐infected tumour cells without restriction by the MHC to limit the development of tumour, and on the other hand, infiltration of NK cells had the strongest correlation with BMP9 expression (Supporting Information Figure S7A), NK cells were selected for treatment. NCG mice were inoculated subcutaneously with HBV‐uninfected cells (HepG2), HBV‐infected cells (HepG2.2.15) or BMP9‐overexpressing HBV‐infected cells (HepG2.2.15‐BMP9). Two weeks later, the mice received an intravenous tail vein injection of 1.0 × 10^7^ NK cells, and IL‐2 was administered intraperitoneally to promote the survival and expansion of NK cells in vivo (Figure 6A). Compared with HepG2.2.15 tumour‐bearing mice, treatment with NK cells exerted better anti‐tumour effects on HepG2 and HepG2.2.15‐BMP9 tumour‐bearing mice (Figure 6B top panel; 6C, D red icon) and a greater number of infiltrating NK cells (Figure 6E, red icon, Supporting Information Figure S7B). Based on these results, BMP9 promotes the infiltration of immune cells into tumour tissues. PD‐1/PD‐L1 blockade has been reported to limit tumour growth by promoting NK cell function.^36^, ^37^ Therefore, we also investigated the therapeutic effect of combination treatment with BMP9 and a PD‐L1 inhibitor. Treatment with anti‐PD‐L1 alone did not alter tumour volume or weight in HepG2, HepG2.2.15 or HepG2.2.15‐ BMP9 tumour‐bearing mice (Figure 6B, bottom panel; 6C, D blue icon); however, compared with treatment with anti‐PD‐L1 alone, the combination treatment with NK cells and anti‐PD‐L1 significantly reduced the tumour volume and weight (Figure 6B, bottom panel; 6C,D green icon) and resulted in greater number of infiltrating NK cells in HepG2 and especially HepG2.2.15‐BMP9 tumour‐bearing mice (Figure 6E, green icon, Supporting Information Figure S7B). Therefore, combination therapy with BMP9 and a PD‐L1‐specific ICB leads to an increase in anti‐tumour activity by NK cells in HBV‐infected HCC.

### 3.7 UTMD‐mediated BMP9 delivery enhances the efficacy of immunotherapy

**FIGURE 7:**
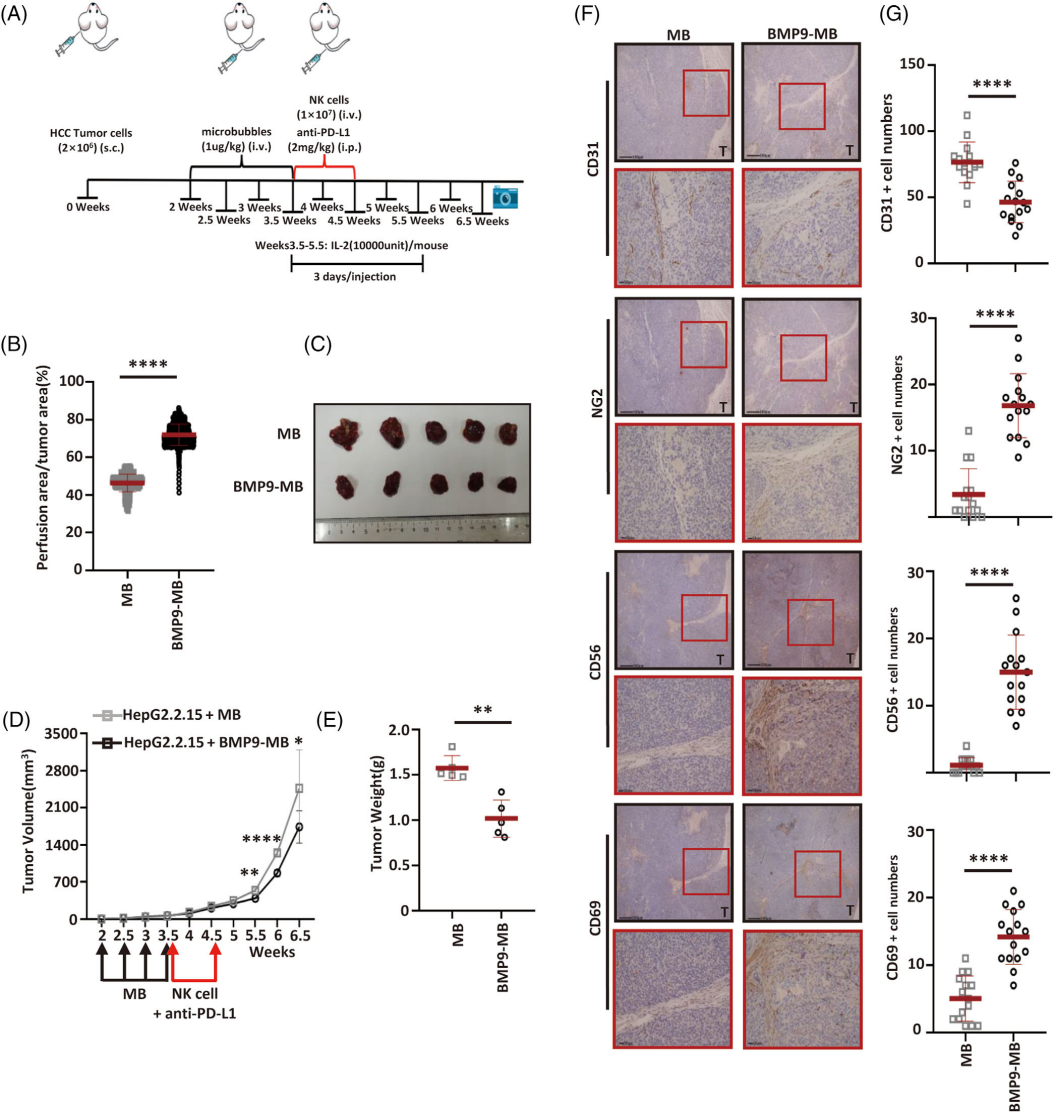
UTMD‐mediated BMP9 delivery improves the efficacy of immunotherapy. (A) Schematic of in vivo studies combining BMP9‐MBs with NK cells and an anti‐PD‐L1 immune checkpoint antibody in a mouse xenograft model established with HepG2.2.15 cells. (B) Quantification of ultrasound detection of perfusion in xenografts formed by HepG2.2.15 cells with or without BMP9‐MB treatment. Mean ± SD, *n* = 694, ****p < .0001, Mann–Whitney U test. (C) Tumour burden of HepG2.2.15 xenografts in animals that were infused with NK cells and the anti‐PD‐L1 immune checkpoint antibody with or without BMP9‐MB treatment after 6.5 weeks. (D,E) Growth curves (D) and tumour weights (E) of HepG2.2.15 xenografts from animals treated with NK cells and the anti‐PD‐L1 immune checkpoint antibody and with or without BMP9‐MBs. Mean ± SD. *n* = 5, *p < .05, **p < .01 and ****p < .0001, Mann–Whitney U test and Student's *t*‐test. (F) Representative images of CD31, NG2, CD56 (NK cell marker) and CD69 (activation marker in NK cell) staining in HepG2.2.15 xenografts from animals that were infused with NK cells and the anti‐PD‐L1 immune checkpoint antibody and treated with or without BMP9‐MBs. (G) Number of CD31, NG2, CD56 and CD69 staining in HepG2.2.15 xenografts from mice that were infused with NK cells and the anti‐PD‐L1 immune checkpoint antibody and treated with or without BMP9‐MBs, as determined by a microscopic analysis of randomly chosen fields at 200× magnification. Mean ± SD. *n* = 15, ****p < .0001, Mann–Whitney U test and Student's *t*‐test.

We used UTMD for the intra‐tumoural delivery of BMP9 combined with the administration of a PD‐L1 inhibitor and NK cells to further explore the clinical application value of BMP9. We first constructed BMP9‐loaded phospholipid‐shelled ultrasound microbubbles composed of a perfluoropropane (C3F8) gas core (BMP9‐MBs) (Supporting Information Figure S8A‐C). BMP9 was released from BMP9‐MBs upon ultrasound stimulation (Supporting Information Figure S8D,E) and prevented the destabilisation of endothelial junctions triggered by HBV‐infected hepatoma cells (Supporting Information Figure S8F). More importantly, BMP9‐MBs do not engage ALK1 but ultrasound releases BMP9 and allows ALK1 engagement (Supporting Information Figure S8G). Mice were inoculated subcutaneously with HBV‐infected cells (HepG2.2.15 cells) to determine the ability of BMP9‐MBs to target the tumour vasculature. Two weeks later, BMP9‐MBs (20 ng/250 μL) were administered by tail vein injection and destroyed in the tumour by ultrasound stimulation (once every 3 days for a total of four times), and then the mice received an intravenous tail vein injection of 1.0 × 10^7^ NK cells and an intra‐abdominal injection of an anti‐PD‐L1 antibody (0.2 mg/mouse). IL‐2 was administered intraperitoneally to promote the survival and expansion of NK cells in vivo (Figure 7A). Compared with HepG2.2.15 tumour‐bearing mice, HepG2.2.15‐BMP9‐MB tumour‐bearing mice, which showed increased tumour vessel perfusion (Figure 7B, Supporting Information Figure S8H,I), exhibited decreased tumour growth after treatment with NK cells and the PD‐L1 inhibitor (Figure 7C‐E). In HepG2.2.15‐BMP9‐MB tumour‐bearing mice, the percentage of NK cells was significantly higher, indicating that UTMD‐mediated BMP9 delivery exerted a strong effect on the infiltration of NK cells (Figure 7F,G). Notably, UTMD‐mediated BMP9 delivery increased the expression of the activation marker CD69 in NK cells, restoring the anti‐tumour function of NK cells when administered in combination with the PD‐L1 inhibitor (Figure 7F,G). These results indicate that UTMD‐mediated BMP9 delivery achieves potential clinical benefits in the treatment of HBV‐infected HCC with immunotherapy.

## 4 DISCUSSION

Cancer immunotherapy based on PD‐1 blocking has changed cancer treatment because it affects more cancer targets and produces longer responses with fewer side effects than other cancer therapies^38^; however, many patients do not respond to this therapy.^39^ PD‐1‐blockade combination therapies have been designed and evaluated in clinical trials to increase the range of responses and enhance their efficacy in patients. One main factor contributing to the poor response is the high rate of abnormal tumour blood vessels.^40^ High levels of tumour blood vessel abnormalities hinder cytotoxic lymphocyte infiltration into tumours and thus, promote tumour escape from immune attack. Moreover, abnormal tumour blood vessels establish a hypoxic microenvironment that induces the expression of immunoregulatory proteins such as PD‐1 and PD‐L1.^41^, ^42^ Conversely, low‐dose TNF‐α in tumour blood vessel normalisation substantially promotes the infiltration of cytotoxic lymphocytes and was thereby shown to improve anti‐tumour vaccination or adoptive T‐cell therapy in a pre‐clinical model of insulinoma.^43^ Tumour blood vessel normalisation also increases the infiltration of cytotoxic lymphocytes into tumours and enhances the efficacy of adoptive cell transfer‐based immunotherapy in B16 melanoma.^44^ In this study, we found that HBV‐infected HCC inhibits cytotoxic lymphocyte infiltration by suppressing BMP9 expression of abnormal vasculature. This result explains the phenomenon that cytotoxic lymphocyte infiltration was enriched in non‐viral‐related HCC compared with HBV‐related HCC.^45^ The use of a UTMD‐mediated BMP9 intra‐tumoural delivery system might improve cytotoxic lymphocyte infiltration by normalising the tumour vasculature, leading to the potentiation of immunotherapy with ICBs.

An abnormal vasculature contributes to tumour progression through impairing perfusion, which results in tumour hypoxia and thereby stimulates angiogenesis to support tumour growth through the induced expression of angiogenic factors. Thus, the response to hypoxia and angiogenesis may reflect an abnormal vasculature. Indeed, angiogenic factors^46^, ^47^, ^48^ and the response to hypoxia^49^ were significantly associated with a higher risk of recurrence after post‐operative and also a major obstacle to improving the prognosis of HCC patients. However, the mechanisms of risk factors and the underlying factors leading to the formation of an abnormal vasculature remain largely unknown. Our RNA‐seq analysis revealed that most genes related to an abnormal vasculature were upregulated in HBV‐infected HCC. Interestingly, the most significantly downregulated gene, *GDF2* (encoding BMP9), was identified. BMP9 has been reported to promote the tumour blood vessel normalisation in lung carcinoma.^17^ In a mouse model of breast cancer, BMP9 deletion increases tumour growth while inhibiting vessel maturation and tumour perfusion.^50^ We initially found that compared with matched adjacent tissues, the BMP9 mRNA and protein levels were significantly decreased in HCC tissues. According to two different GEO datasets, *BMP9* mRNA expression levels were downregulated in tumours and inversely correlated with the tumour grade. This evidence suggests that BMP9 has the potential to inhibit the abnormal vasculature of HBV‐infected HCC. In East Asia, the majority of HCC develops is caused by HBV infection in an environment of chronic inflammation. HCC is a hypervascularized tumour, and there is an increasing evidence indicated that HBV stimulation of angiogenesis may lead to malignancy of HCC. Since we found that BMP9 expression was significantly associated with HBV infection, we suspect that HBV infection was the underlying cause of the suppressed BMP9 expression. HBV is a DNA virus that can be incorporated into the host genome, and this integration itself may result in signal pathway aberration within the host cells, DNA methylation and histone modification.^51^ We reduced the HBV DNA load with TFV, which is a very potent and effective nucleotide analog that inhibits HBV,^25^ to further confirm the relationship between HBV infection and BMP9 expression. When the expression of HBV DNA was suppressed in HBV‐infected hepatoma cells, BMP9 expression increased. In addition, BMP9 was significantly downregulated in tumour cells isolated from HBV‐infected HCC tissues compared with those isolated from HBV‐uninfected HCC tissues. Based on these results, the downregulation of BMP9 in HCC is induced by HBV infection.

In human cancers, depending on the cancer type, different functions for BMP9 have been described. In prostate^52^ and breast cancer,^50^ BMP9 exerts tumour‐suppressive activity, since it inhibits migration, growth and invasion, whereas in osteosarcoma^53^ and ovarian cancer,^54^ it acts to promote proliferation. In addition, BMP9 as a metastasis suppressor in ovarian cancer was also demonstrated.^55^ The biological role of BMP9 in HCC remains unclear. Jae Woo Jung et al.^56^ found that BMP9 was expressed at lower levels in liver cancer tissues than in normal liver tissues based on gene expression data from normal and tumour tissue databases. Supporting this finding, we also observed lower BMP9 mRNA levels in HCC cells than in normal liver cells from two different GEO datasets for HCC, and the decreased expression of BMP9 in HCC was further validated by IHC staining of clinical specimens. In contrast, Qi Li^56^ and Blanca Herrera^57^ reported a tendency toward an increase in BMP‐9 expression in HCC tissue compared to adjacent tissue. This paradox is probably because different laboratories use different rules for classifying the semi‐quantitative scores for the BMP‐9 status. Previous studies have illustrated the dual role of BMP9 in proliferation is dependent on the HCC cell type.^58^ In addition, physiological levels of BMP9 (2‐5 ng/mL) promote EpCAM+ cancer stem cell (CSC) properties in Huh7 cells,^59^ but high levels (200 ng/mL) inhibit CD44^+^ CSC properties in Hep3B cells.^58^ The role of BMP9 in tumour angiogenesis also depends on the endothelial cell type. On the one hand, BMP9 has been shown to inhibit both VEGF‐ and FGF‐induced angiogenesis in bovine aortic endothelial cells, HUVECs and human dermal microvascular endothelial cells *in vitro*,^60^ indicating that BMP9 is an anti‐angiogenic factor. On the other hand, BMP9 has been shown to promote mouse embryonic stem cell‐derived endothelial cell angiogenesis in vitro and tumour‐associated endothelial cell proliferation in a human pancreatic cancer xenograft model.^61^ BMP9 and TGF‐β both belong to the TGF‐β superfamily. More and more evidence suggests that the TGF‐β and BMP pathways play an opposing role, and one pathway enhancement is accompanied by another pathway weakening in the progression of some cancers including HCC.^29^ In our study, BMP‐9 also played an opposite role than TGF‐β in vasculature formation. We think that the main reason to determine the role of TGF‐β and BMP pathways in vasculature formation is context‐dependent factors. BMP‐9 binds to ALK‐1 with high affinities,^62^ inducing p‐Smad1/5/8 signaling,^29^ and leads to the maturation and stabilisation of newly formed blood vessels, being implicated in arteriovenous specification and pericyte recruitment.^30^ However, TGF‐β binds to ALK‐5, inducing p‐Smad2/3 signalling, and leads to the inhibition of endothelial cell proliferation. ALK5 indeed has been reported to increase TGF‐β‐induced endothelial cells permeability and actin cytoskeleton remodeling.^63^ Our results confirmed that BMP9 can inhibit the Rho‐ROCK‐MLC pathway by p‐Smad1/5/8 in regulation of vascular normalisation of HBV‐infected HCC. Some BMPs, such as BMP4^64^ and BMP7,^65^ have been reported to be associated with higher activity of the Rho‐ROCK signalling, but some experimental results have clearly supported that BMP9 mediated a unique inhibitor function to Rho‐ROCK signalling^66^ in vascularizing properties, which is consistent with our findings. This different function could also be explained by previous studies demonstrating a BMP9 role in extracellular matrix protein deposition and remodeling.^67^

In this study, we revealed another mechanism underlying the anti‐angiogenic effect of BMP9 in tumour blood vessel normalisation in HCC. Although our in vitro assays support a direct inhibitory effect of BMP9 on the abnormal tumour vasculature, we cannot exclude the pro‐growth effect of BMP9 on our in vivo tumour model, in which increased tumour growth was observed for BMP9‐expressing HepG2.2.15 cells (Figure 6C). Indeed, although physiological levels of BMP9 (1‐10 ng/mL) might prevent the destabilisation of endothelial junctions (Supporting Information Figure S7C), we did not observe an increase in the proliferative ability of HepG2.2.15 cells after stimulation with physiological levels of BMP9 (Supporting Information Figure S7D). This result is consistent with a previous report on HepG2 cells.^68^ One explanation for this observation is that the more normal the vasculature becomes with BMP9 treatment, the more oxygen and nutrients the tumour will have, which is supported by our findings that increased tumour vessel perfusion coincided with decreased levels of tumour hypoxia in BMP9‐overexpressing HBV‐infected HCC xenografts. Nevertheless, in our in vivo animal model, the combination of treatment with NK cells and anti‐PD‐L1 significantly reduced tumour growth, suggesting that the increase in tumour growth induced by BMP9 is only a temporary phenomenon occurring in the process of immunotherapy.

Despite enormous theoretical and experimental support for the benefit of vascular normalisation in HCC immunotherapy, effectively achieving this normalisation in the clinic remains complex and daunting. Although initial anti‐angiogenic strategies primarily targeted VEGF and appear to only transiently normalise and subsequently prune vessels,^69^ the features of vascular normalisation (pericyte coverage and tumour perfusion) were eventually lost and replaced by pronounced vascular regression.^70^ Studies of murine and human tumours have identified the onset of normalisation, typically 1−2 days after commencement of therapy, followed by an eventual ‘closure’ of the normalisation window, at which point features of normalisation are lost.^71^ Genetic approaches to normalisation, such as promoting endothelial cell quiescence (e.g., PHD2 knockdown^72^) and enhancing vascular function (e.g., RGS5 knockdown^73^), give rise to a more prolonged normalisation phenotype, in the absence of dramatic vessel regression. In our study, the features of vascular normalisation (pericyte coverage and tumour perfusion) are still in the high range, either in BMP9‐overexpressing HBV‐infected HCC xenografts (Figure 4) or in the intratumourally delivered BMP9 treatment (Figure 6), which is different from VEGF inhibitors. Previous studies have shown the ability of BMP9 to induce vascular normalisation in Lewis lung carcinoma^17^ and breast cancer,^50^ but the method of overexpressing BMP9 in tumour cells is not suitable for clinical cancer immunotherapy, as it is often associated with serious problems such as immunogenicity and cytotoxicity.^74^ In addition, similar to most drugs with clinical application value, BMP9 has certain limitations when administered systemically. First, because BMP9 has a short plasma half‐life, the plasma BMP9 concentration fluctuates constantly. Second, after an intravenous injection, BMP9 is distributed throughout the body and does not accumulate in the tumour. Therefore, the administration's approach should be modified. UTMD has emerged as a novel non‐viral gene delivery technique because of its safety, high efficiency and potential for local transfer.^75^ An increasing number of pre‐clinical studies have successfully documented the therapeutic benefits of the UTMD in the delivery of chemotherapeutic drugs to various malignant tumours. The encapsulation of chemotherapeutic drugs in microbubbles and subsequent local release and absorption in the target tissue through ultrasonic stimulation may help to improve the therapeutic effects.^76^ In the present study, we provide experimental support for the efficacy and therapeutic potential of UTMD combined with BMP9 for the treatment of HBV‐infected HCC in vivo. We were able to target BMP9 accurately without any obvious toxicity due to the specificity and efficacy of this delivery method. Even so, what we still need to note is that although the ultrasound microbubble mainly distributes in the reticuloendothelial system, such as the liver, spleen, lungs and brain, UTMD is not as useful in the brain or entire abdomen. Because masses can sometimes be tiny and far from the skin's surface. It is also limited in some parts of the body because the sound waves cannot go through air (such as in the lungs) or through bone. Therefore, UTMD‐mediated BMP9 delivery is of subcutaneous tumour in the liver, gallbladder, pancreas, breast, ovarian, kidneys, bladder and prostate.

**FIGURE 8:**
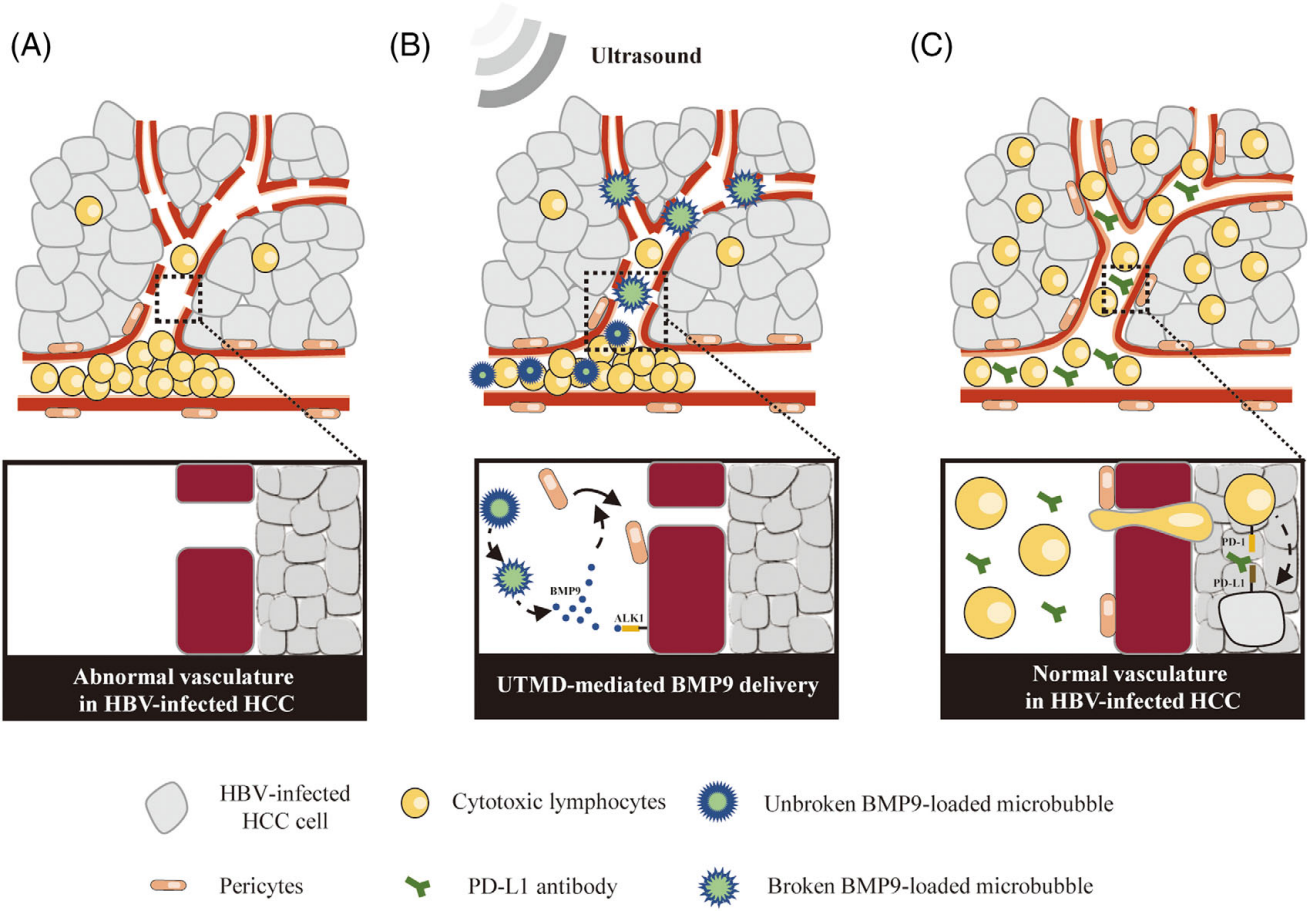
Working model illustrating the strategy for improving the efficacy of immunotherapy with UTMD‐mediated BMP9 delivery in HBV‐associated HCC. (A) Vascular abnormalities inhibit the intratumoural infiltration of cytotoxic lymphocytes in HBV‐infected HCC. (B) UTMD‐mediated BMP9 delivery restores pericyte recruitment and endothelial junctions. (C) The normal vasculature supports the therapeutic efficacy of cytotoxic lymphocytes administered in combination with a PD‐L1 inhibitor and restores anti‐tumour function.

In conclusion, our study reveals a novel molecular mechanism by which HBV promotes abnormal tumour blood vessel function in HBV‐associated HCC. Downregulation of BMP9 by HBV activated the Rho/ROCK/MLC signalling pathway to induce abnormal tumour blood vessel formation. Furthermore, UTMD‐mediated BMP9 delivery restored the anti‐tumour function of NK cells administered in combination with a PD‐L1 inhibitor to suppress tumour growth in an HBV‐infected HCC xenograft model. BMP9, an important factor for tumour vasculature normalisation, represents a potential therapeutic target to reinvigorate immunotherapy for the treatment of HBV‐associated HCC (Figure 8).

## CONFLICT OF INTEREST STATEMENT

All authors give consent for the publication of the manuscript in Journal of Experimental & Clinical Cancer Research. The authors have no conflict of interest to declare.

## Supporting information

Supporting Information

## Data Availability

The data that support the findings of this study are available from the corresponding author upon reasonable request.
