# Supplementary material for: BMP9‐induced vascular normalisation improves the efficacy of immunotherapy against hepatitis B virus‐associated hepatocellular carcinoma
Source: Clin Transl Med. 2023 May 2;13(5):e1247. doi: 10.1002/ctm2.1247 (PMC10154878; doi:10.1002/ctm2.1247)
Supplement: Supplementary file 1 — Supporting Information [file CTM2-13-e1247-s001.docx]

**BMP9-induced vascular normalization improves immunotherapy in hepatitis B virus-associated hepatocellular carcinoma**

Yulong Han, QiuzhongPan, Zhixing Guo, Yufei Du, Yingying Liu, Jingjing Zhao, Jinfeng Xu, Jieying Yang, Dijun Ouyang, Yan Tang, Qijing Wang, Yongqiang Li, Jia He, Mengjuan Yang, Yue Huang, Hao Chen, Chaopin Yang, Xinyi Yang, Jinqi You, Fengze Sun, Yuanyuan Chen, Yan Tang, Minghao Ren, Yao Zhu, Jianchuan Xia, and Tong Xiang

**Table of contents**

Supplementary materials and methods

Supplementary table

Supplementary figures and figure legends

**Antibodies and chemicals.** Antibodies specific for the following targets and the following reagents were used in this study: BMP9 (PA5-11931, Invitrogen, 1:100 for immunohistochemistry (IHC); ab207318, Abcam, 1:1,000 for western blotting (WB)), hepatitis B surface antigen (HBsAg; ZM0122, Zhongshan Jinqiao Bio. Co. for IHC), hepatitis B core antigen (HBcAg; ZA0121, Zhongshan Jinqiao Bio. Co. for IHC), mouse CD31 (77699, Cell Signaling Technology, 1:100 for IHC and NB100-1642AF405, NOVUS, 1:100 for immunofluorescence (IF)), mouse α-SMA (56856S, Cell Signaling Technology, 1:200 for IHC and IF), mouse NG2 (MAB6689, R&D Systems, 1:200 for IHC and IF), VEGFR2 (9698S, Cell Signaling Technology, 1:800 for IHC), human CD31 (ZA0568, Zhongshan Jinqiao Bio. Co. for IHC), human α-SMA (ZM0003, Zhongshan Jinqiao Bio. Co. for IHC), human CD8 (ZA0508, Zhongshan Jinqiao Bio. Co. for IHC), human CD57 (ZM0058, Zhongshan Jinqiao Bio. Co. for IF), fluorescein isothiocyanate (FITC)-dextran (40 kDa) (4009, Chondrex), DyLight 594 *Lycopersicon esculentum* (tomato) lectin (FL-1177-1, Vector Labs), phospho-MLC (Ser19) (3671T, Cell Signaling Technology, 1:1,000 for WB), ROCK1 (ab134181, Abcam, 1:500 for WB), ROCK2 (ab125025, Abcam, 1:500 for WB), RhoA (ab187027, Abcam, 1:5,000 for WB), ALK1(ab183332, Abcam, 1:2,000 for WB), phospho-smad1/5/8(Ser463/465) (sc12353, Santa Cruz Biotechnology, 1:1,000 for WB), phospho-smad3(Ser423/425) (ab52903, Abcam, 1:1,000 for WB), smad6 (ab80049, Abcam, 1:2,000 for WB), GAPDH (60004-1-1g, Proteintech, 1:5,000 for WB), human CD56 (MA511563, Invitrogen, 1-2 µg/mL for IHC), human CD69 (ab233396, Abcam, 1:500 for IHC), Alexa Fluor 647 (A-21244, Invitrogen, 4 µg/mL for IHC). A Hypoxyprobe^TM^ plus kit (HP2-100Kit) was purchased from Hypoxyprobe, and tenofovir (TFV; S1401) was purchased from Selleckchem. ALK1 inhibitor (HY-12274) was purchased from MedChemExpress (MCE), smad1/5/8 inhibitor ([LDN193189](https://www.medchemexpress.cn/LDN193189.html), HY-12071) was purchased from MedChemExpress (MCE). ROCK Activator (4-methyl-2-oxopentanoic acid, HY-W012722) was purchased from MedChemExpress (MCE), Rho Activator I (CN01) was purchased from Cytoskeleton, and a RhoA Activation Assay Kit (80601) was purchased from Wuhan NewEast Biosciences Co., Ltd. An InVivoMab anti-human PD-L1 (B7-H1) antibody (be0285) was purchased from BioXCell, DSPE-PEG 2000 and DSPC were purchased from Avanti Lipids (Alabaster, USA), and NK Cell (QLS-010) was purchased from Shandong Qilu Stem Cell Engineering Co., Ltd.

**Evaluation of the effect of HBV on tumor growth.** To observe the effect of HBV on tumor growth, 2.5 × 10^6^ HepG2 and HepG2.2.15 cells were inoculated subcutaneously into the right flank of 4-week-old female NCG mice. The mice were purchased from GuangDong GemPharmatech Co., Ltd. At the end of the experiment, the mice were euthanized, and paraffin tumor tissue sections were immunostained following the manufacturer’s instructions. To evaluate antiviral drug efficacy, 2.5 × 10^6^ HepG2.2.15 cells were inoculated subcutaneously into the right flank of female NCG mice. When the tumors reached 100–200 mm^3^, the mice were randomly divided into two groups. One group received physiological saline (100 µl/mouse injected intraperitoneally (i.p.) every two days), and the other group received tenofovir (30 mg/kg injected i.p. every two days). Tumor size was measured every 7 days. At the end of the experiment, the mice were euthanized, and paraffin tumor tissue sections were immunostained following the manufacturer’s instructions.

**Multiplex immunohistochemical analyses of tumor vessels.** Xenograft mouse tissues were fixed with formalin, embedded in paraffin and subjected to IHC. Briefly, embedded tissues were dewaxed and hydrated, and the samples underwent high-temperature antigen repair. A primary antibody was incubated at room temperature for 1 h. Then, a corresponding specific secondary antibody and signal amplification solution were incubated at room temperature for 10 min. After washing with PBS, high-temperature antigen repair was performed at a pH required for other antibodies, and the above steps were repeated until all antibodies were applied. Finally, cell nuclei were stained with DAPI, and anti-quenching sealing tablets were used to seal the slides. The sections were imaged with Polaris (Akoya Biosciences).

**Radiotherapy.** A radiotherapy sensitivity test was performed with mice inoculated subcutaneously in the left flank with HepG2.2.15 cells and in the right flank with HepG2.2.15-BMP9 cells. At 28 days after subcutaneous inoculation, one group of mice received 2 Gy irradiation of the tumors in the right and left armpits, whereas the other group of mice with tumors in both armpits did not. Then, at 7 days post-irradiation, the mice were euthanized, and the tumors were harvested, weighed and photographed.

**Evans Blue-Albumin permeation assay.** An Evans Blue-Albumin permeation assay was performed to detect changes in permeability *in vitro*. HepG2, HepG2.2.15, HepG2.2.15-BMP9, Huh6, HB611 and HB611-BMP9 cells were cultured in six-well plates in EndoGRO^TM^ medium, and HUVECs were seeded in the upper cavity of a 0.1% gelatin-coated Transwell insert (0.4 µm). After the HUVECs were cocultured with the HCC cells for 4 days, Evans Blue-bound albumin was added to the upper chamber for 90 min. By measuring the optical density (620 nm) of the medium collected from the two chambers, the ratio of Evans Blue-Albumin between the upper and lower chambers was measured and used to detect the change in permeability.

**Migration assay.** We first collected the culture supernatants of HepG2, HepG2.2.15, HepG2.2.15-BMP9, Huh6, HB611 and HB611-BMP9 cells. Each supernatant was added to the lower chamber of a Transwell system, and then transferred MBVPs or HBVPs were cocultured in Transwell inserts (5,000 cells/well; 8 µm; Corning) in pericyte medium (ScienCell). The cell lines and MBVPs or HBVPs were cocultured for 24 h, and then the number of migrating cells was statistically analyzed. In another experiment, we collected the culture supernatants of cell lines (HepG2.2.15, HepG2.2.15, and HepG2.2.15-BMP9 cells with a Rho or ROCK activator; and HB611, HB611-BMP9 and HB611-BMP9 cells with a Rho or ROCK activator), and then transferred MBVPs or HBVPs were cocultured in Transwell inserts (5,000 cells/well; 8 µm; Corning) in pericyte medium (ScienCell). The cell lines and MBVPs or HBVPs were cocultured for 24 h, and then the number of migrating cells was statistically analyzed.

**RNA-seq sample preparation and sequencing.** Total RNA was isolated and purified using TRIzol according to the manufacturer's protocol. The RNA library was then sequenced using an Illumina HiSeq4000 at GENE DENOVO Co., Ltd. (Guangzhou, China). Using STAR (v2.5.1b), RNA-seq reads were aligned with a reference genome. A specific approach was used to control the false discovery rate. Genes with an adjusted p value (padj) < 0.05 and a fold change of 2 were defined as exhibiting significant differential expression. HTSeq v0.6.0 was used to convert the comparative results to obtain RNA-seq gene expression measurement results, which are reported as fragments per kilobase of exon per million fragments mapped (FPKM).

**Real-time quantitative PCR analysis.** HBV DNA was isolated according to standard genomic DNA isolation methods. First, at 65°C, HBV-infected cells were lysed for 4 h in lysis buffer (50 mM Tris–HCl, pH 8.0; 50 mM EDTA; 100 mM NaCl; 1% SDS) containing protease K (200 μg/ml). A DNA extraction kit (Magen) was used to isolate HBV DNA from the lysed cells. One microgram of isolated HBV DNA was included in a 20-µl mixture using 40 units of plasmidsafe DNase (Epicentre Technologies #E3101K, Madison, WI, USA) and digested for 8 h at 37°C. After the digestion, the isolated HBV DNA was inactivated at 70°C for 30 min. Two microliters were extracted from the 20-µl reaction volume for real-time PCR. HBV DNA detection primers were used for real-time quantitative PCR analysis. Total RNA was isolated using TRIzol reagent (Invitrogen) according to the manufacturer's instructions. GoScript Reverse Transcriptase (Promega) was used to synthesize first-strand cDNA. RT–PCR was performed using the following gene-specific primers:

BMP9-F: GCCCTTCTTTGTTGTCTTCT

BMP9-R: GACTGCTCTCACCTGCCTCT

HBV DNA-F: ATGGAGAA CACAACATCAGG

HBV DNA-R: GAGGCATAGCAGCAGGATG

GAPDH-F: ACATCGCTCAGACACCATG

GAPDH-R: TGTAGTTGAGGTCAATGAAGGG

**IHC.** Clinical HCC tumor tissues and xenograft mouse tissues were fixed with formalin, embedded in paraffin and subjected to IHC. Briefly, embedded tissues were dewaxed and hydrated, and the samples underwent high-temperature antigen repair. A primary antibody was incubated at 4°C overnight. After incubation with a specific secondary antibody the next day, a DAB peroxidase substrate kit (Dako) was used. After DAPI (Invitrogen) was used to stain cell nuclei, neutral resin was used for sealing and imaging. Semiquantitative IHC scoring was performed as follows: 0, cancer cell staining area of less than 1%; 1, cancer cell staining area of 2-25%; 2, cancer cell staining area of 26-50%; 3, cancer cell staining area of 51-75%; and 4, cancer cell staining area of greater than 75%. The staining intensity was scored as follows: 0, no staining; 1, weak staining; 2, moderate staining; and 3, strong staining. Each piece of tissue was sectioned consecutively and stained twice, and the sections were reviewed by two different pathologists. The staining index (values 0–12) was obtained by multiplying the staining intensity by the semiquantitative score. To identify quantitation of the number of angiogenic vessel and pathological vessel normalization, CD31, α-SMA and VEGFR2 were calculated by image J-Angiogenesis analyzer.

**Western blot analysis.** Cells were lysed in SDS-loading buffer, followed by SDS–PAGE and transfer to PVDF membranes (Millipore). Primary antibodies were incubated and combined with specific HRP-conjugated secondary antibodies, and enhanced chemiluminescence was used to visualize the protein bands (ECL, Yeasen).

**IF analyses**. Mouse tissue samples were immediately frozen in OCT compound (for 5-µm serial sections). After rewarming, the sections were washed with PBS. The sections were immersed in 0.2% Triton X-100, incubated for 5 min, and then washed with PBS again. The sections were incubated with a primary antibody at room temperature for 1 h and then with Alexa Fluor 647-conjugated goat anti-rabbit IgG (Invitrogen) for 1 h. Then, the nuclei were stained with DAPI (Invitrogen), and anti-quenching sealing tablets were used to seal the slides, which were imaged with a confocal laser-scanning microscope (Olympus FV1000). The integrated optical density (IOD) was calculated with ImageJ software, and the mean IOD (MOD) was calculated as the IOD/area according to the ratio to quantify the IF staining intensity.

**Synergistic immunotherapy.** Identification of therapeutic effects after vascular normalization, 2.5 × 10^6^ HepG2, HepG2.2.15-HBV and HepG2.2.15-BMP9 cells were inoculated subcutaneously in the female NCG mice right flanks. After inoculated subcutaneously 14 days, each type of cells was divided into four groups. One group was used as control, one group was treated with NK cells (1 × 10^7^ i.v.), the other group was treated with anti-PD-L1 (bioxcell USA; 2 mg/kg i.p.), and the last group was treated with NK cells and anti-PD-L1 in combination. NK cell treatment group was injected with IL-2 (10,000 U, once every 3days, seven times in total), and the other two groups were injected with equal volume of normal saline. Mice were imaged once a week using In vivo Xtreme II imager (Bruker), and checked periodically for health status. At the end of the experiment, mice were euthanized, and tumors were harvested and weighed. Tissue samples were immediately fixed in 4% PFA overnight at 4 °C, dehydrated and embedded in paraffin (for 5 µM serial sections).

Identification of therapeutic effects after construct a BMP9-loaded microbubbles which promoted vascular normalization and synergistic immunotherapy, 2.5 × 106 HepG2, HepG2.2.15-HBV and HepG2.2.15-BMP9 cells were inoculated subcutaneously in the female NCG mice right flanks. One group was used as control, and the other group was treated with NK cells and anti-PD-L1 in combination. After inoculated subcutaneously 14 days, One group was used as control that blank microbubbles (250µl, i.v.) was administered injection every 3 days for a total of 4 times; the other group which MBs-BMP9 (BMP9-loaded microbubbles) (20ng/250µl, i.v.) was administered injection every 3 days for a total of 4 times. Two groups were all treated with NK cells (1 × 107 i.v.) and anti-PD-L1 (bioxcell USA; 2 mg/kg i.p.) in combination. Two groups were also injected with IL-2 (10,000 U, once every 3days, four times in total). Checked periodically for health status. At the end of the experiment, mice were euthanized, and tumors were harvested and weighed. Tissue samples were immediately fixed in 4% PFA overnight at 4 °C, dehydrated and embedded in paraffin (for 5 µM serial sections).

**Supplementary table**

| **Variable** | **Univariate cox** | | **Multivariate cox** | | |
| --- | --- | --- | --- | --- | --- |
|  | ***P*-value** | **HR(95％CI)** | ***P*-value** | | **HR(95％CI)** |
| **Age**  ≥50 or<50 | 0.888 | 0.869(0.377-2.002) |  | |  |
| **Gender**  Male or female | 0.884 | 0.95(0.38-2.372) | |  |  |
| **HBsAg**  Negative or positive | 0.254 | 3.001(0.405-2.225) |  | |  |
| **AFP**  ≤400ng/ml or＞400ng/ml | 0.568 | 1.256(0.569-2.772) |  | |  |
| **TNM stage**  I or II/III | **0.034** | 1.498 (1.016-2.207) | **0.048** | | 2.193(1.007-4.776) |
| **liver cirrhosis**  Yes or no | 0.140 | 0.251(0.034-1.859) |  | |  |
| **BMP9 expression**  High or low | **0.016** | 0.294(0.101-0.858) | **0.027** | | 0.299(0.102-0.875) |

**Supplementary figures and figure** **legends**


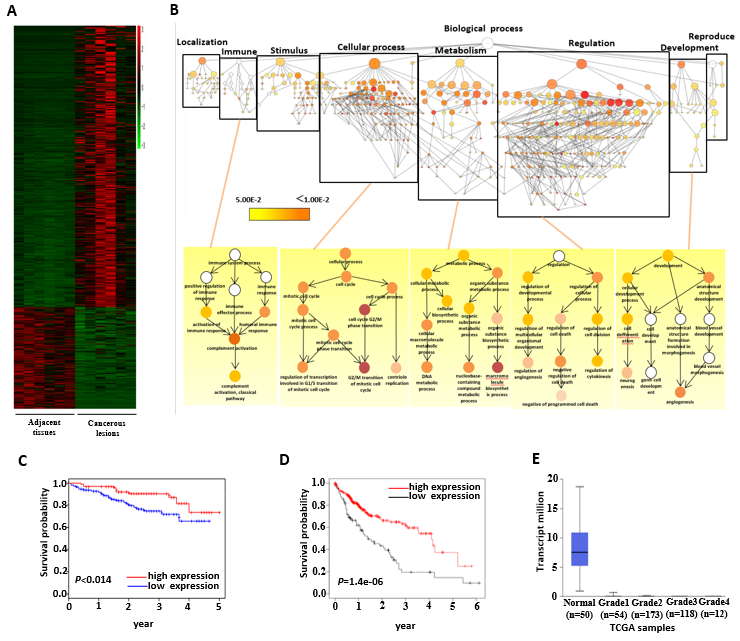


**Supplementary Figure 1. Enrichment of all differentially expressed genes by HBV infection.** **A.** FPKM values of all differentially expressed genes between HBV-infected tumor tissues and adjacent noncancerous tissues. Padj<0.05. **B.** GO tree obtained from BiNGO showing the hierarchy of biological processes related to immune response, cell cycle, metabolic process, developmental process and angiogenesis between HBV-infected tumor tissues and paired adjacent noncancerous tissues (p < 0.05). White nodes (p > 0.05) indicate the relationship between upstream and downstream nodes. **C.** HCCDB datasets for HCC (http://lifeome.net/database/hccdb/search.html) showed that lower mRNA levels of BMP9 were associated with poorer overall survival, p<0.05. **D.** KM plotter datasets for HCC (http://kmplot.com/analysis/index.php?p=service&cancer=liver_rnaseq) showed that lower mRNA levels of BMP9 were associated with poorer overall survival, p<0.01. **E.** UALCAN datasets showed that BMP9 expression was consistently downregulated across all grades of HCC (<http://ualcan.path.uab.edu/analysis.html>).


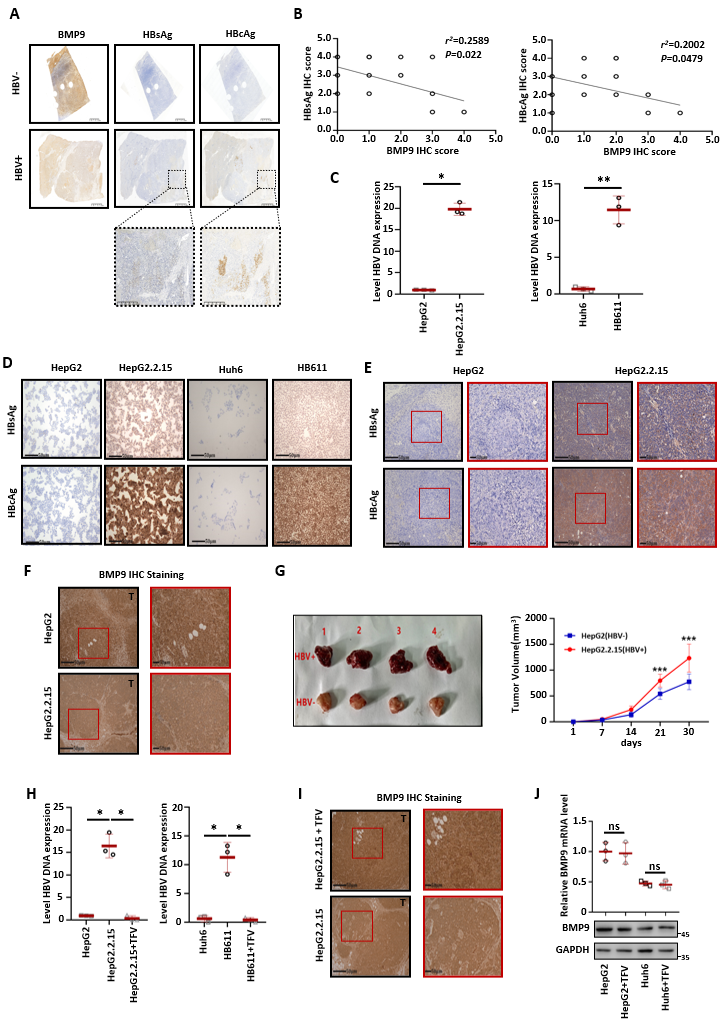


**Supplementary Figure 2. Viral HBcAg and HBsAg verification and TVF efficacy verification. A.** BMP9, HBsAg and HBcAg expression analyzed by IHC in HCC patients infected or uninfected with HBV virus**. B.** Correlation between HBV antigen (HBsAg or HBcAg) and BMP9 expression by Spearman correlation analysis. n=10. **C.** HBsAg expression in paired HBV-infected and HBV-uninfected hepatoma cell lines (HepG2.2.15 vs. HepG2 cells and HB611 vs. Huh6 cells). Mean ± SD. n=3, *p < 0.05, **p < 0.01, Mann–Whitney U test and Student's t test. **D.** The content of viral HBcAg and HBsAg was assessed in paired HBV-infected and HBV-uninfected hepatoma cell lines (HepG2.2.15 vs. HepG2 cells and HB611 vs. Huh6 cells) by IHC. **E.** IHC confirmation of the content of viral HBcAg and HBsAg in paired HBV-infected and HBV-uninfected hepatoma cell line xenografts (HepG2.2.15 vs. HepG2 xenografts). **F.** BMP9 expression was analyzed in HepG2 and HepG2.2.15 xenografts by IHC. **G.** HBV - and HBV + hepatoma cell lines (HepG2 vs. HepG2.2.15) were injected subcutaneously into mice to observe the tumor grow in vivo. Means ± SD, n=4, ***p＜0.001, Dunnett’s t test. **H.** Real-time PCR confirmed the inhibition of HBsAg content by TFV in HBV-infected hepatoma cell lines *in vitro*. Mean ± SD. n=3, *p < 0.05, Kruskal–Wallis H test. **I.** BMP9 expression analyzed by IHC in HepG2.2.15 and HepG2.2.15 xenografts treated with TFV. TFV: tenofovir. T: tumor. **J.** Relative BMP9 mRNA (upper panel) and protein (lower panel) levels in HBV-uninfected hepatoma cells treated with TFV. Means ± SD, n=3, ns: not significant, Dunnett’s t test.


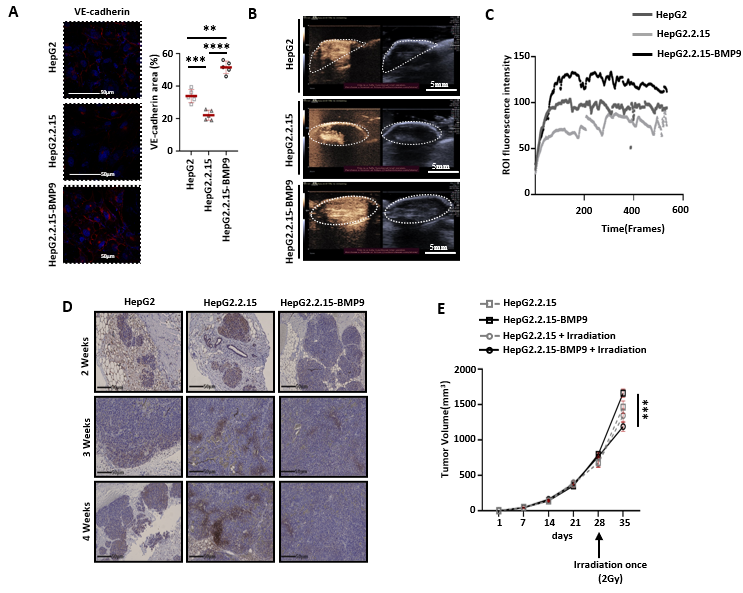


**Supplementary Figure 3. Overexpression of BMP9 inhibits the abnormal vasculature in HBV-infected HCC. A.** Representative images (left panel) and quantification (right panel) of VE-cadherin staining to assess extent of endothelial junction in HUVECs cocultured in the presence of different HCC cell supernatant. The VE-cadherin area/percentage of total area was analyzed to determine extent of endothelial junction by performing a microscopic analysis of randomly chosen fields at 600× magnification. Means ± SD, n=5, ***p < 0.001 and ****p < 0.0001, Dunnett’s t test. **B.** Representative images of ultrasound (US) and [contrast-enhanced ultrasound](https://pubmed.ncbi.nlm.nih.gov/29423461/) (CEUS) detection of the perfusion area of xenografts formed by HepG2, HepG2.2.15 or HepG2.2.15 cells overexpressing BMP9. **C.** The region of interest (ROI) fluorescence intensity area/tumor total area was determined to assess the perfusion efficiency of the tumor vasculature. **D.** Representative images of antihypoxia probe staining of xenografts formed by HepG2, HepG2.2.15 or HepG2.2.15 cells overexpressing BMP9. **E.** Growth curves of xenografts formed by the HepG2 and HepG2.2.15 cell lines following radiotherapy (2 Gy). Mean ± SD, ***p < 0.001, Student's t test.


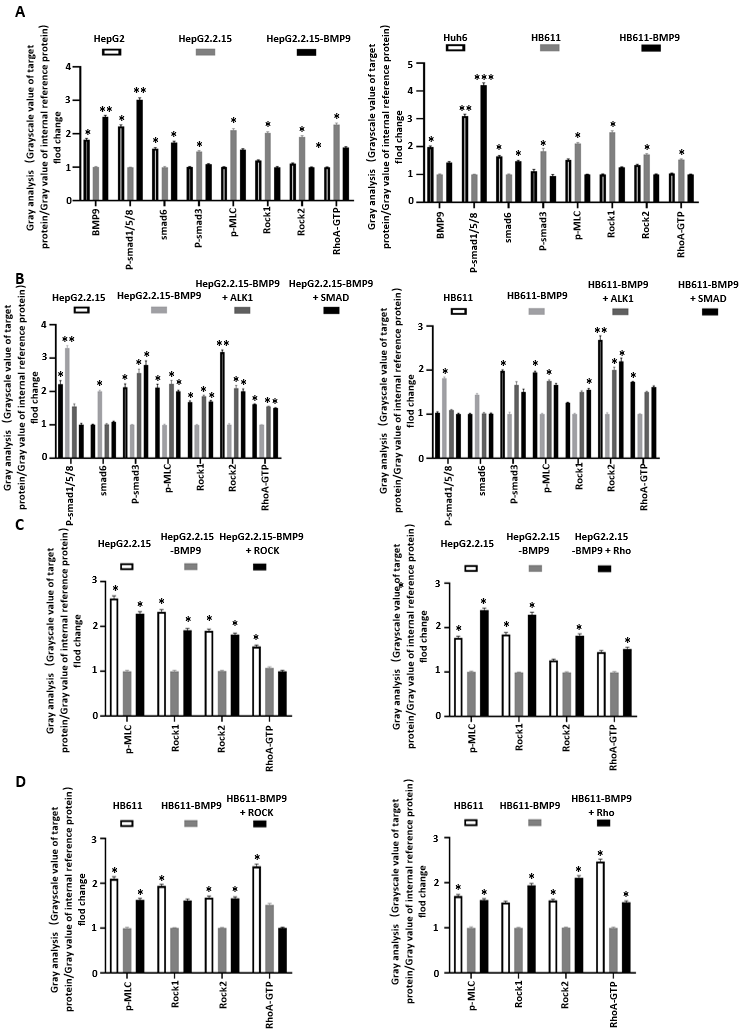
 **Supplementary Figure 4. Gray analysis of western blot results.** Grayscale value of target protein/Gray value of internal reference protein determined based on image J- set Measurements analysis. **A.** Signaling pathway molecules protein expression in different cell lines (HepG2, HepG2.2.15 or HepG2.2.15 cells overexpressing BMP9 and Huh6, HB611 or HB611 cells overexpressing BMP9). Mean ± SD, n=3, *p < 0.05, **p < 0.01, ***p < 0.001, Kruskal–Wallis H and Student's t test. **B.** Signaling pathway molecules protein expression in different cell lines (HepG2.2.15 or HepG2.2.15 cells overexpressing BMP9 and HB611 or HB611 cells overexpressing BMP9) after added ALK1 and smad1/5/8 inhibitors. Mean ± SD, n=3, *p < 0.05, **p < 0.01, ***p < 0.001, Kruskal–Wallis H and Student's t test. **C.** Signaling pathway molecules protein expression in different cell lines (HepG2.2.15 or HepG2.2.15 cells overexpressing BMP9) after added ROCK and Rho activators. Mean ± SD, n=3, *p < 0.05, **p < 0.01, ***p < 0.001, Student's t test. **D.** Signaling pathway molecules protein expression in different cell lines (HB611 or HB611 cells overexpressing BMP9) after added ROCK and Rho activators. Mean ± SD, n=3, *p < 0.05, **p < 0.01, ***p < 0.001, Student's t test.


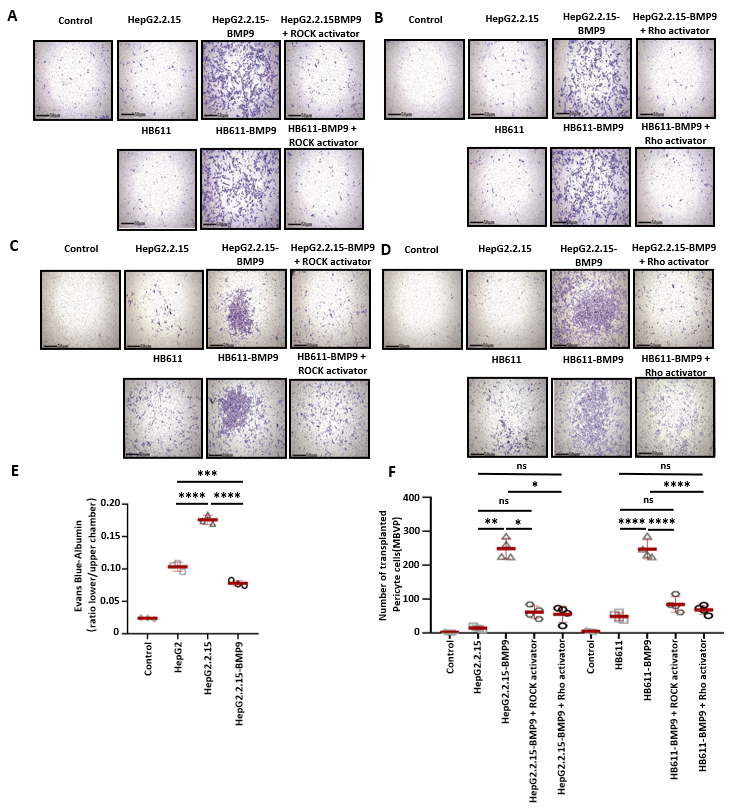


**Supplementary Figure 5. BMP9 promotes the normalization of the tumor vasculature via the Rho/ROCK/MLC axis. A and B.** Representative image of [human brain vascular pericyte](https://www.ixcellsbiotech.com/product/animal-primary-cells/mouse-brain-vascular-pericytes-mbvp#:~:text=Cultured%20primary%20mouse%20BVP%20%28MBVP%29%20are%20a%20useful,a%20wide%20variety%20of%20central%20nervous%20system%20diseases.) (HBVP) migration induced by HBV-uninfected, HBV-infected and HBV-infected hepatoma cell lines after Rho/ROCK activator stimulation. **C and D.** Representative image of mouse [brain vascular pericyte](https://www.ixcellsbiotech.com/product/animal-primary-cells/mouse-brain-vascular-pericytes-mbvp#:~:text=Cultured%20primary%20mouse%20BVP%20%28MBVP%29%20are%20a%20useful,a%20wide%20variety%20of%20central%20nervous%20system%20diseases.) (MBVP) migration induced by HBV-uninfected, HBV-infected and HBV-infected hepatoma cell lines after Rho/ROCK activator stimulation. **E.** Evans Blue-Albumin permeation through the upper mouse endothelial monolayer in the lower chamber containing HepG2 or HepG2.2.15 cells to quantify the integrity of the endothelial monolayer. Mean ± SD, n = 3, ***p < 0.001, ****p < 0.0001, Dunnett’s t test. **F.** Quantification of [mouse brain vascular pericyte](https://www.ixcellsbiotech.com/product/animal-primary-cells/mouse-brain-vascular-pericytes-mbvp#:~:text=Cultured%20primary%20mouse%20BVP%20%28MBVP%29%20are%20a%20useful,a%20wide%20variety%20of%20central%20nervous%20system%20diseases.) (MBVP) migration toward HBV-uninfected, HBV-infected and HBV-infected hepatoma cell lines after Rho/ROCK activator stimulation. Mean ± SD, n = 4, ns: not significant, *p < 0.05, **p < 0.01, ****p < 0.0001, Kruskal–Wallis H test and Dunnett’s t test.


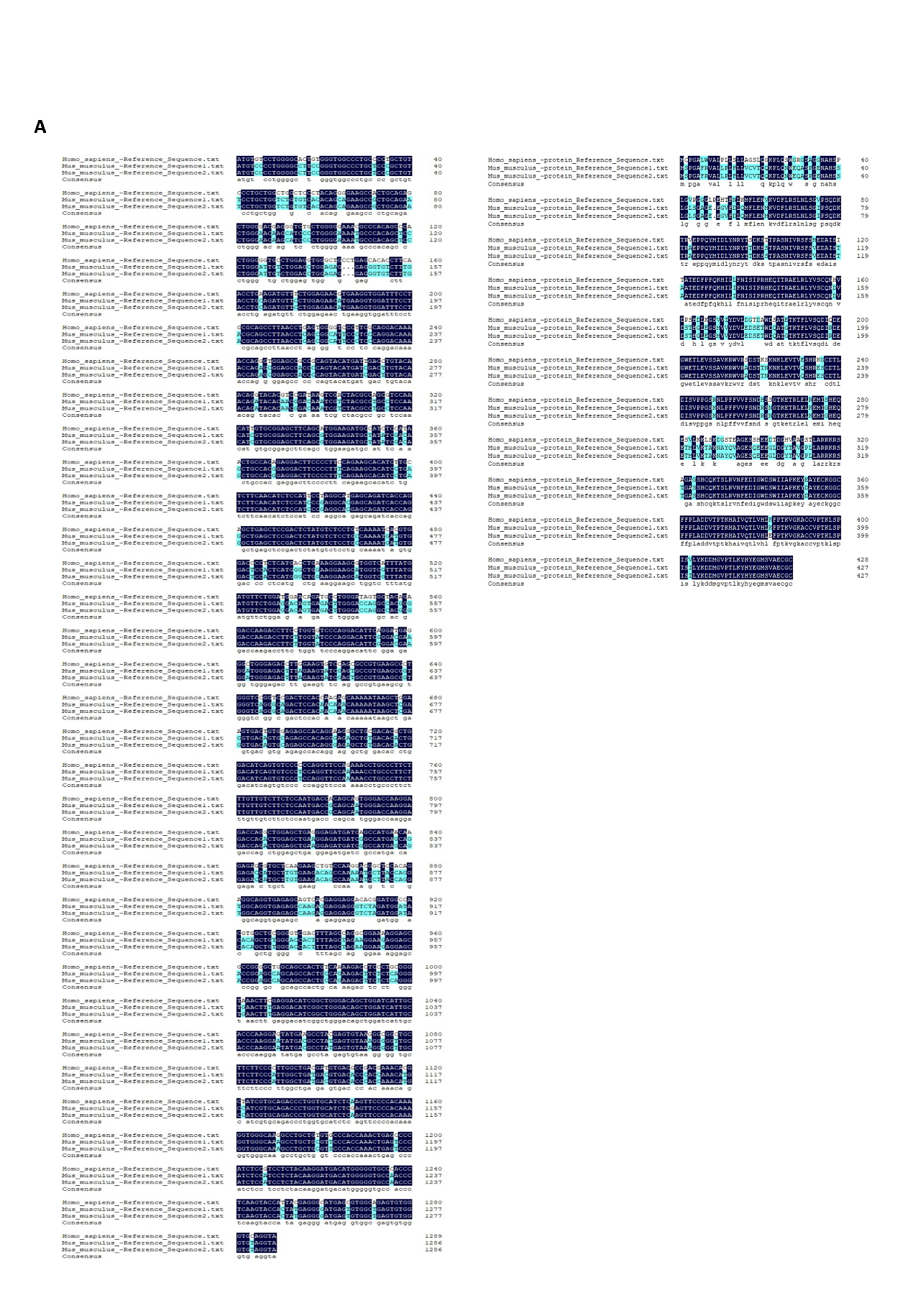


**Supplementary Figure 6. Amino acid and protein sequence alignments of BMP9 among species.** **A.** The software Dnaman was used to compare the BMP9 amino acid sequences (left) of different species, and the software Translate (<https://web.expasy.org/translate/>) was used to compare the BMP9 protein sequences (right) of different species.


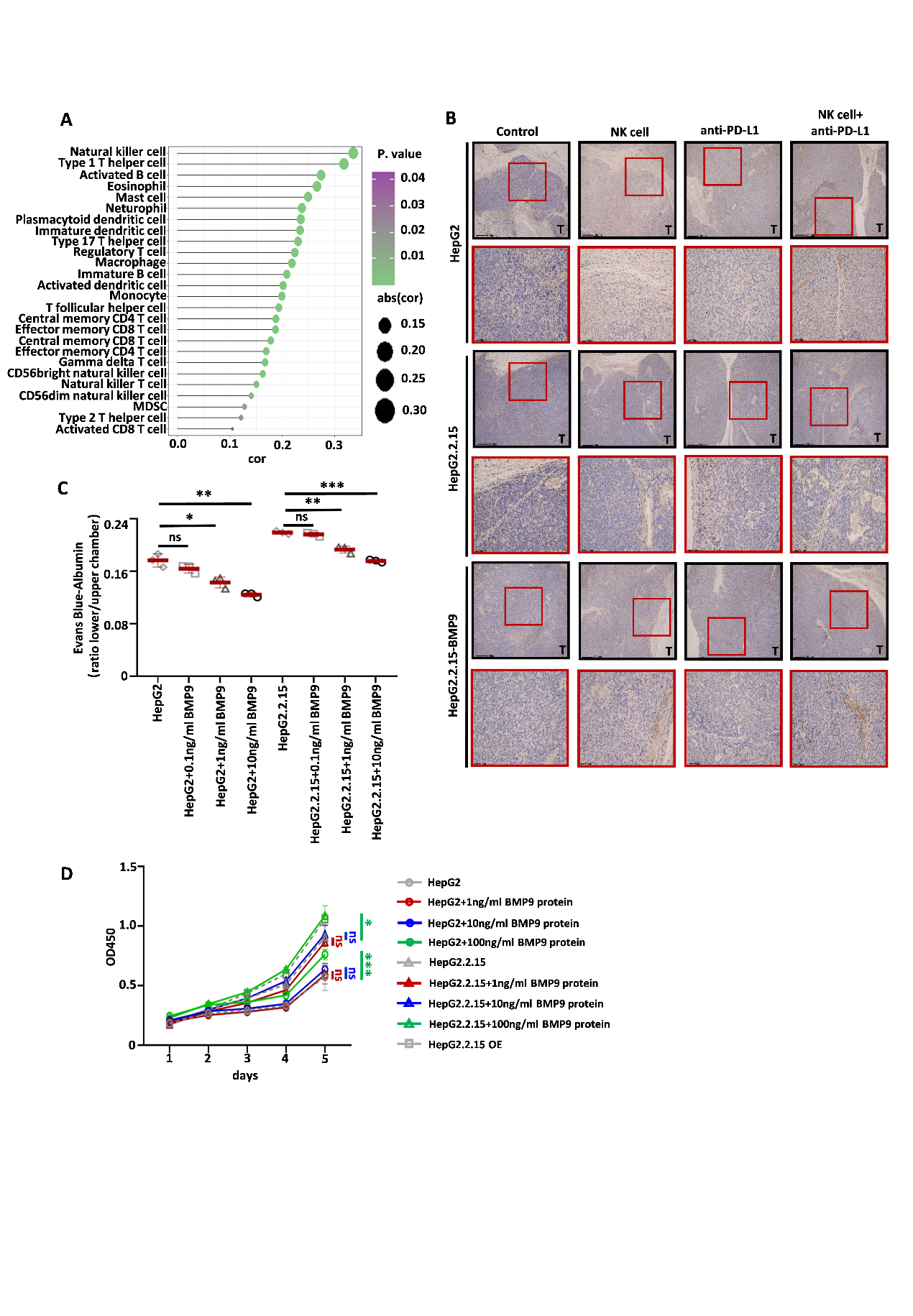


**Supplementary Figure 7. BMP9 expression promotes changes in the quantity of infiltrating NK cells in the context of PD-L1 blockade *in vivo*. A.** The correlation between BMP9 expression and immune cells infiltration from TCGA-LIHC dataset ([https://www.cancer.gov](https://www.cancer.gov/)) using Rstudio analysis. Tumor infiltrated immune cells was calculated by CIBERSORT and GSVA software package (<https://pubmed.ncbi.nlm.nih.gov/23323831/>). **B.** Representative images of infiltrating NK cells in xenografts derived from HepG2, HepG2.2.15 or HepG2.2.15 cells overexpressing BMP9 that were infused with NK cells and/or an anti-PD-L1 immune checkpoint antibody. **C.** Quantification of Evans Blue-Albumin permeation through the upper endothelial monolayer in the lower chamber containing HCC cell lines stimulated with different concentrations of the BMP9 protein. Mean ± SD, n=3, ns: not significant, *p < 0.05, **p < 0.01, ***p < 0.001, Student's t test. **D.** Proliferation of HCC cell lines treated with different concentrations of the BMP9 protein as determined with CCK-8. Mean ± SD, n=6, ns: not significant, *p < 0.05, ***p < 0.001, Kruskal–Wallis H test.


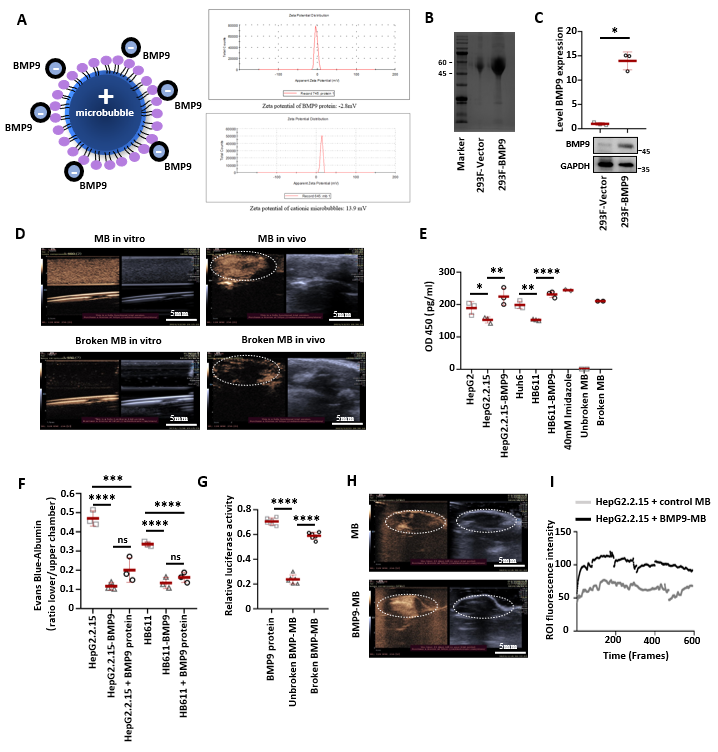


**Supplementary Figure 8. Preparation and verification of ultrasound-targeted microbubbles (UTMs). A.** Schematic diagram of BMP9-loaded phospholipid-shelled microbubbles (left) and determination of BMP9 protein and microbubble charge levels (right). **B**. Coomassie brilliant blue staining results for BMP9 expressed by 293F cells. **C.** Relative BMP9 mRNA and protein levels measured by real-time PCR and western blot analyses of 293F cells. Mean ± SD, n=3, *p < 0.05, Mann–Whitney U test. **D.** Representative image of ultrasound (US) and [contrast-enhanced ultrasound](https://pubmed.ncbi.nlm.nih.gov/29423461/) (CEUS) detection of ultrasound-targeted microbubble (MB) destruction (UTMD) *in vitro* and *in vivo*. **E.** The concentration of BMP9 released from different hepatoma cell lines and BMP9-MBs with or without UTMD. Mean ± SD, n=3, *p < 0.05, **p < 0.01, ****p < 0.0001, Student's t test. **F.** Evans Blue-Albumin permeation assay performed with different hepatoma cell lines with or without BMP9 stimulation. Mean ± SD, n=3, ns: not significant, ***p < 0.001, ****p < 0.0001, Dunnett’s t test. **G.** A dual-luciferase reporter experiment confirmed the binding of BMP9 to endothelial cells. Mean ± SD, n=6, ****p < 0.0001, Student's t test. **H.** Representative images of ultrasound detection of perfusion in xenografts formed by HepG2.2.15 cells with or without BMP9-MB treatment. **I.** The region of interest (ROI) fluorescence intensity area/tumor total area was determined to assess the perfusion efficiency of the tumor vasculature in HepG2.2.15 xenografts with or without BMP9-MB treatment.
